# Hydralazine Revives Cellular and Ocular Lens Health-Span by Ameliorating the Aging and Oxidative-Dependent Loss of the Nrf2-Activated Cellular Stress Response

**DOI:** 10.3390/antiox12010140

**Published:** 2023-01-06

**Authors:** Bhavana Chhunchha, Eri Kubo, Ronald R. Krueger, Dhirendra P. Singh

**Affiliations:** 1 Department of Ophthalmology and Visual Sciences, University of Nebraska Medical Center, Omaha, NE 68198, USA; 2 Department of Ophthalmology, Kanazawa Medical University, Kanazawa 9200293, Japan

**Keywords:** aging, oxidative stress, antioxidants, Nrf2, peroxiredoxin 6, hydralazine, age-related diseases

## Abstract

A major hallmark of aging-associated diseases is the inability to evoke cellular defense responses. Transcriptional protein Nrf2 (nuclear factor erythroid-derived 2-related factor) plays a pivotal role in the oxidative stress response, cellular homeostasis, and health span. Nrf2’s activation has been identified as a therapeutic target to restore antioxidant defense in aging. Here, we demonstrated that FDA-approved drug, hydralazine (Hyd), was a reactivator of the Nrf2/ARE (antioxidant response element) pathway in various ages and types of mouse (m) or human (h) lens epithelial cells (LECs) and mice lenses in-vitro/in-vivo. This led to Hyd-driven abatement of carbonyls, reduced reactive oxygen species (ROS), and reduced 4-HNE/MDA-adducts with cytoprotection, and extended lens healthspan by delaying/preventing lens opacity against aging/oxidative stress. We elucidated that Hyd activated the protective signaling by inducing Nrf2 to traverse from the cytoplasm to the nucleus and potentiated the ARE response by direct interaction of Nrf2 and ARE sequences of the promoter. Loss-of-function study and cotreatment of Hyd and antioxidant, N-acetyl cysteine (NAC) or Peroxiredoxin (Prdx)6, specified that Nrf2/ARE-driven increase in the promoter activity was Hyd-dependent. Our study provides proof-of concept evidence and, thereby, paves the way to repurposing Hyd as a therapeutic agent to delay/prevent aging and oxidative-related disorders.

## 1. Introduction

Preservation of redox homeostasis is central to maintaining cellular integrity and physiological function. An imbalance between oxidation and reduction phenomenon can lead to cell/tissue damage [1]. This imbalance can emerge from endogenous and exogenous stresses as well as deterioration of the cellular antioxidant defense pathway during aging [2,3,4], which is accompanied by cellular damage and cell death. Indeed, disruption of antioxidant response and, thereby, increased oxidative load has been shown to be a major cause for various aging-related pathologies, including blinding diseases [2,5,6,7,8,9]. To cope with the pathogenic effects generated by oxidative stressors, cells have evolved antioxidant defense mechanisms to encounter these stresses to maintain redox homeostasis [2,10,11]. Studies have shown that cellular oxidant/antioxidant equilibrium is maintained in a dynamical way via threshold regulation of cellular antioxidants’ expression [12]. Exposure of mammalian cells to cellular and environmental stressors generally initiates antioxidant transcriptional responses, which are mainly up-regulated through a master transcription factor, Nrf2 (nuclear factor erythroid-derived 2-related factor) [13,14]. This biological phenomenon results in the coordinated increased expression of phase II antioxidant genes, such as glutathione S-transferase (GSTπ), catalase (Cat), glutathione-peroxidase (GPxs), heme oxygenase 1 (HO1), glutamate-cysteine ligase subunits (GCLC and GCLM), NAD(P)H, quinone oxidoreductase 1 (NQO-1), and peroxiredoxins (Prdxs). However, when these protective antioxidants are dysregulated, as mostly observed during aging, cell activity is deranged with increase in cell death due to ROS amplification [2,10,11,15,16]. To maintain cell/organ health, redox balance is strictly maintained and fine-tuned by prevention, interception, and repair via the regulatory mechanism driven master switches, such as Nrf2/Keap1 (Kelch-like ECH-associated protein 1). It is reported that reactivation of Nrf2 activity in aging cells by means of electrophile(s) or by metformin (anti-aging drug), restores aging-associated abnormalities [3,10,17,18,19]. Recently, FDA-approved drug hydralazine (Hyd) has been shown to be cytoprotective, and its application extends health/life span. Interestingly, the beneficial activity of Hyd has been found to be attributed to its activation of the Nrf2/ARE antioxidant pathway [20,21,22]. These studies suggest that repression of Nrf2-dependent antioxidative response is a key contributor to aging-associated etiopathologies and can be corrected by means of potent Nrf2 activators, such as Hyd [3,18,19,20,21,22,23,24].

Many increasing age-related pathologies or diseases share common risk factor(s). With advancing age, oxidant generation from several environmental and cellular sources is increased, while, at the same point, the Nrf2/ARE-mediated antioxidant protective pathway, the primary line of defense, is repressed. Studies have demonstrated that aging-related amplification of ROS-driven oxidative damage and accumulation of intracellular oxidized proteins aggregates occur due to loss of antioxidant expression and lesser capacity to mitigate oxidative load [11,24,25,26]. Additionally, ample studies, including our own work, reveal that decline in the Nrf2/ARE antioxidant response in response to oxidative stressors leads to damage of aging cells [6,19,26,27,28,29]. Thus, central to our understanding, activation, and suppression of antioxidants, responsible for cytoprotecting capacity, is dependent on Nrf2 integrity and cellular abundance. The Nrf2/ Keap1 pathway plays a critical role in maintaining cellular antioxidant defense against oxidative, electrophilic, and cellular stress. Nrf2, a cap‘n’collar basic-region leucine zipper transcription factor, regulates more than 200 genes, including a battery of antioxidant genes, to maintain cellular physiology and resuscitate cell health [16,30,31,32,33,34,35]. Additionally, recent studies, specifically those using Nrf2-deficient mice, as disease modes, support Nrf2’s pivotal role in the protection and prevention against various pathologies [36,37]. It has been shown that electrophiles-mediated increased lifespan or healthspan is related to Nrf2-dependent cellular responses [38,39,40,41,42,43]. The levels of Nrf2 are largely regulated through somatic loss-of-function of Keap1 in response to electrophiles or oxidants, which leads to Nrf2’s translocalization to the nucleus, where it binds to ARE sequences and upregulates the target protective genes. Keap1, an adaptor protein of cullin 3-based E3 ubiquitin ligase, serves as a sensor for electrophilic reagents and oxidative stress [35,44]. In addition to repression of Nrf2 activity through Keap1 under basal conditions, glycogen synthase kinase-3 (GSK-3) has been demonstrated to phosphorylate selective serine residues in the Neh6 domain of Nrf2, generating a degradation domain, which is identified through the ubiquitin ligase adapter β-transducin repeat containing protein (β-TrCP) and then targeted for proteasomal degradation independent of Keap1 [45,46,47].

Nevertheless, the major molecular event underlying deranged physiological functions and failure of cellular homeostasis in aging-related diseases is chronic elevation of ROS-driven oxidative stress due to unresponsiveness protective antioxidant response [15,48,49,50]. Thus, pharmacological targeting of the Nrf2 antioxidant pathway to reactivate antioxidant response is of interest in connection to halt oxidants-induced pathological signaling. By using electrophiles, such as curcumin, sulforaphane, as well as the anti-aging drug, metformin, we have demonstrated that aging cells are responsive, and the cells display increased expression and activity of Nrf2/ARE antioxidants, leading to attenuation of cell death and the onset of lens opacity [3,17,19,51]. This convinced us that pharmacological interventions should be an extremely helpful and better research tool. In addition, invention of protective pharmacological reagent(s) can be a more time-restricted intervention with a dosage-dependent way, as well as easily deliverable to subjects, than genetic intervention (DNA/protein delivery). Additionally, we think that pharmacological drug(s) will be an asset to identifying pathogenic factor(s) and pathways that occur during the onset of aging-related pathologies. In this scenario, based on our previous study and others showing how oxidative signaling is accelerated due to loss of Nrf2 antioxidant system and how this contributes to the onset of aging-associated diseases [2,10,19,30,31], we postulated that reactivation of the Nrf2 antioxidant pathway might be a promising strategy to prevent oxidative/aging-induced pathologies [18,23].

The eye is a prime target for environmental stress and highly exposed to oxidative inductors, sunlight, or pollutants. Compared to other tissues, the ocular lens contains a potent antioxidant system to combat oxidative stress-induced damage. However, with advancing age, deterioration of the antioxidant system results in higher oxidative load-driven failure of the lens’ homeostasis and onset of lens opacity [3,51,52,53,54,55,56,57,58,59]. Herein, it is worth mentioning that the eye lens has been suggested as one of the best model systems to delineate the molecular mechanism(s) of oxidative- and age-associated pathological signaling as well as therapeutic interventions [3,60,61,62]. It is intriguingly conceivable that aging and oxidative stress are common denominators for development of aging disorders, including age-related cataracts, and, therefore, discovery of therapeutic reagents using the lens as a model system to screen drug activity and the mechanism of action can be applied to treat or prevent other aging diseases. Furthermore, considering the pivotal roles of protein modifications, such as carbonylation and 4-HNE and MDA-adducted proteins, which increase with aging, and their injurious contributions in the development of age-related diseases, including ARC [30,63,64,65,66,67,68], it would be considerable to choose a water soluble and nontoxic drug with a potent activator of Nrf2/ARE antioxidant pathway to develop a promising therapeutic strategy of reversing the injurious signaling causing aging pathologies. In this regard, we identified a known Nrf2/ARE inductor, Hyd, having carbonyl scavenging and antiaging properties [21,22,68,69,70,71,72], to test its protective potential in abating lens opacity. However, therapeutic potential and the molecular mechanism(s) of Hyd in preventing or protecting eye lens/LECs in response to aging or oxidative stress-mediated pathobiology has not been illustrated.

In this study, therefore by using a variety of LECs of variable ages as well as in-vivo Hyd-treated mouse lenses facing oxidative stress ex-vivo as model systems and assessments, we addressed the effect and efficacy of Hyd in restoring aging/oxidative stress-related adverse signaling causing pathobiology of the eye lens. We found that the Nrf2 antioxidant pathway acts as a driver mechanism in aging/oxidative-related signaling. We identified that Hyd treatment corrected impaired transcriptional activity of Nrf2/ARE in aging LECs and lenses in-vivo as well as prevented oxidative stress-induced lens opacity ex-vivo. Thus, our finding showed that Hyd instillation in the eye (in the form of eye drops) resulted in increased cellular protection and extended the healthspan of eye lenses and LECs (prevention or delay of lens opacity). Altogether, our studies demonstrated that repression of the Nrf2-mediated antioxidative response is a plausible key player of the initiation or progression of etiopathologies during aging and oxidative stress. Finally, our findings build a point that therapies aimed at Nrf2 antioxidant pathway reactivation by Hyd should have the promising potential to protect or prevent cell/organ damage leading to aging/oxidative-related disease conditions.

## 2. Materials and Methods

### 2.1. Cell Culture

#### 2.1.1. SRA-hLECs (Human Lens Epithelial Cells)

Human LECs cell line ((SRA01/04) immortalized with SV40 [73], a kind gift from late Dr. Venkat N. Reddy, Oakland University, Rochester, MI, USA) [73] were maintained routinely in our laboratory. Briefly, cells were cultured in a 100 mm tissue culture plate in Dulbecco’s Modified Eagle Medium (DMEM, Invitrogen, Waltham, MA, USA) supplemented with 15% heat-inactivated fetal bovine serum (FBS, Atlanta Biologicals, Atlanta, GA, USA), 100 µg/mL streptomycin, and 100 µg/mL penicillin in an incubator maintained at 37 °C with 5% CO_2,_ as described previously [5,74]. In the paper text, we will designate the immortalized LECs as SRA-hLECs. For the experimentation, SRA-hLECs were plated the day before treatment, and cells attaining 60–70% confluency were treated with nontoxic concentrations of hydralazine (Hyd).

#### 2.1.2. Isolation of LECs from the Lenses of Human Subjects and Maintenance

Primary hLECs were isolated from normal eye lenses of deceased persons or healthy donors of different ages (22 years (y), 23 y, 25 y, 55 y, 57 y, 59 y, 69 y, 68 y, 67 y, 70 y old) received from the Lions Eye Bank, Nebraska Medical Center, Omaha, NE, and National Development and Research Institute (NDRI), Inc., Philadelphia, PA, USA. Primary LECs isolated from these lenses were used for the present study. According to regulation HHS45CFR 46.102(f), studies involving lenses or LECs from deceased subjects are not considered human subject(s) research as stated under 45CFR46.102(f) 10(2) and, therefore, do not require IRB oversight. Primary LECs were isolated from the lenses as described previously [3,51,54,75,76]. In brief, cultured explants were trypsinized, and isolated cells were subcultured in petri dishes containing complete medium. Cell cultures showing 90 to 100 percent confluency were harvested and used for the study [74,77,78]. αA-crystallin, a specific marker, was used for validation of LECs’ identity.

#### 2.1.3. Generation of Mouse Lens Epithelial Cells (mLECs) and Primary mLECs

All animal studies followed the recommendations set forth in the “Statement for the Use of Animals in Ophthalmic and Visual Research” by the Association for Research in Vision and Ophthalmology and were approved by the Institutional Animal Care and Use Committee (IACUC), University of Nebraska Medical Center (UNMC), Omaha, NE. LECs were isolated from C57BL/6 mice and maintained in DMEM with 10% FBS, as described earlier [51,74,76,79]. Like SRA-hLECs, mLECs were cultured in 100 mm plate and next-day treated with Hyd (diluted in the complete culture media) at 70% confluency.

C57BL/6 mice of 6 months (M), 16M, and 22 M were purchased from Charles River Laboratories (Wilmington, MA, USA) and were maintained at a stable temperature and humidity under specific pathogen-free conditions in a cleaned animal facility, as described earlier [3,31]. Primary mLECs were isolated from lenses and cultured in collagen IV precoated dishes containing complete DMEM media (15% FBS). At 90–100% confluency, cells were harvested and used for the study.

### 2.2. Cell Viability Assay

Cell growth or H_2_O_2_-mediated toxicity was analyzed using MTS assay. In brief, cell viability of Hyd (Millipore Sigma, Rockville, MD, USA)-treated or untreated LECs facing H_2_O_2_-induced oxidative stress was evaluated using a colorimetric MTS assay (Promega, Madison, WI, USA) according to the manufacturer’s method and as described previously [6,80,81]. This assay of cell proliferation/viability uses 3-(4,5-dimethylthiazol-2-yl)-5-(3-carboxymethoxyphenyl)-2 to (4-sulphophenyl) 2H-tetrazolium salt. At the end of predefined incubation, MTS dye was added. After 2 h of incubation, the absorbance was read at 490 nm with a plate reader (DTX 880, Multimode detector, Spectra MAX Gemini, San Jose, CA, USA). The data obtained were normalized with absorbance of the untreated control(s).

### 2.3. Measurement of Reactive Oxygen Species (ROS)

Intracellular ROS levels of LECs facing H_2_O_2_-driven stress in absence or presence of Hyd were assessed with H_2_-DCF-DA (2′-7′-dichlorodihydrofluorescein diacetate) dye, a non-fluorescent fluorescein derivative as described in our published report [3,31]. On the day of experiment, the medium was discarded, and cell culture wells were washed with PBS and incubated in the same buffer containing 10 µM of H_2_-DCF-DA dye for 30 min at 37 °C. After diffusion into the cells, H_2_-DCF-DA is deacetylated by cellular esterase into a non-fluorescent compound that was later oxidized by ROS into 2′-7′-dichloroflurescein (DCF). ROS levels were quantified at Ex485 nm/Em530 with a Spectra Max Gemini EM (Molecular Devices, San Jose, CA, USA).

To measure the intracellular redox state levels of lenses isolated from Hyd-administered C57BL/6 mice eye, lenses were carefully isolated and immediately frozen at −80 °C. The intracellular levels of ROS were quantified by H_2_-DCF-DA according to our published protocol [2,3,31]. Briefly, lenses were homogenized (100 mg/mL) in freshly prepared homogenization buffer (50 mM phosphate buffer containing 0.5 mM PMSF, 1 mM EDTA, 1 µM pepstatin, 80 mg/L trypsin inhibitor, pH 7.4). The same amount of homogenate was added to 96-well cell culture plates. To measure the levels of ROS, H_2_-DCF-DA (30 µM final concentration) dye was added and incubated for 30 min. Intracellular fluorescence was detected at O.D., Ex485 nm/Em530, with a Spectra Max Gemini EM (Molecular Devices, San Jose, CA, USA).

### 2.4. Measurement of the Oxidative Effect by Lipid Peroxidation Assay

Lipid peroxidation (LPO) assay is based on the reaction of N-methyl-2-phenylindole, a chromogenic reagent with MDA (malondialdehyde) and 4-HNE (4-hydroxyalkenals) at 45 °C. Either MDA or 4-HNE reacts with two molecules of N-methyl-2-phenylindole to yield a stable chromophore with maximal absorbance at 586 nm. LPO assay in LECs/lenses exposed with H_2_O_2_ in absence or presence of Hyd was carried out according to manufacturer’s instructions (Lipid Peroxidation Microplate Assay Kit; Oxford Biomedical Research, Rochester Hills, MI, USA) and our published report [11,80]. Total cell lysate was prepared from LECs/lenses, and equal amounts of protein were used for the assay. Absorbance was measured at 586 nm using a plate reader (DTX 880, Multimode detector, Spectra MAX Gemini, San Jose, CA, USA).

### 2.5. Protein Carbonyl Assay

The protein carbonyl content was measured in LECs/lenses exposed to H_2_O_2_ in absence or presence of Hyd using Protein Carbonyl Content Assay Kit (MAK094, Sigma-Aldrich, Saint Louis, MO, USA) following the company’s protocol. The protein content of LECs (samples) were determined using the BCA assay. Samples were treated with 10% streptozocin solution to remove interfering nucleic acid. Purified water served as background control. Dinitrophenylhydrazine (DNPH) solution was added to each sample and incubated for 10 min, and then 87% TCA solution was added to the sample. Supernatant was removed by high centrifugation, and ice-cold acetone was added to each pellet and was placed for 30 s in a sonication bath and then further incubated at −20 °C for 5 min. Carefully, acetone was removed from each pellet by high centrifugation, and 6M guanidine solution was added to the pellet and sonicated. An amount of 100 µL of each sample was transferred to the 96-well plate, and reading was measured at 375 nm (absorbance) with a plate reader (DTX 880, Multimode detector, Spectra MAX Gemini, San Jose, CA, USA).

### 2.6. Measurement of 8-Hydroxydeoxyguanosine

DNA is a biologically significant target of oxidative stress. The generation of 8-Hydroxydeoxyguanosine (8-OHdG) is a ubiquitous marker of oxidative stress among numerous types of oxidative DNA damage. We measured the levels of 8-OHdG in LECs/lenses exposed to H_2_O_2_ with or without Hyd treatment using The OxiSelect™ Oxidative DNA Damage Elisa kit (STA-320, Cell Biolabs, INC., San Diego, CA, USA). To prepare a standard curve, different concentrations (0–20 ng/mL) of 8-OHdG standard was prepared by diluting in assay diluent as mentioned in the company’s protocol. In brief, genomic DNA was isolated from LECs/lenses treated with H_2_O_2_ in absence or presence of Hyd and converted to single stranded DNA. DNA samples were digested with nuclease P1 (Catalog No. N8630, Millipore Sigma, Rockville, MD, USA) followed by the incubation with 10 units of alkaline phosphatase (Catalog No. P5931, Millipore Sigma, Rockville, MD, USA) at 37 °C for 1 h in 100 mM Tris, pH 7.5. The 8-OHdG standards (50 µL) or samples (50 µL) were added to the well of the 8-OHdG conjugate coated plate and incubated at room temperature (RT) for 10 min with shaking. Thereafter, diluted anti-8-OHdG (50 µL) antibody was added to each well and incubated for 1 h on an orbital shaker at RT. The wells were washed, and diluted secondary antibody-enzyme conjugate (100 µL) was added and incubated on a shaker for 1 h at RT. After washing the wells, 8-OHdG activity was recorded by the addition of 100 µL warm substrate solution. Enzymatic reaction was stopped by the addition of 100 µL of stop solution, and absorbance was measured at 450 nm with a microplate reader (DTX 880, Multimode Detector, Molecular device, San Jose, CA, USA).

### 2.7. Nrf2/ARE Driven Luciferase Reporter Assay

SRA-hLECs, mLECs, or different ages of primary hLECs were transfected with pRBGP2 (3x ARE-LUC) containing three ARE sites or its mutant pRBGP4 (3x mut ARE- LUC) plasmids, a kind gift from Dr. Hozumi Motohashi, Japan) [82] along with *Renilla*, pRL-TK vector (Promega, Madison, WI, USA) using Neon transfection system (Invitrogen, Waltham, MA, USA). After 14 h, cells were washed and treated with Hyd for 24 h. Luciferase activity was measured using Dual-Glo luciferase assay system with 96-well plate (Promega, Madison, WI, USA) submitted to a microplate reader (DTX 880, Multimode Detector, Molecular device, San Jose, CA, USA) [3].

### 2.8. Protein Isolation and Expression Analysis

Protein expression was examined by Western blot analysis as described previously [3,7,18,20,23]. In brief, SRA-hLECs or mLECs/lenses treated with Hyd for different time periods were processed for protein extraction using ice-cold radioimmune precipitation buffer (RIPA buffer). Cellular extract containing equal amounts of protein (measured by the Bradford method) from each sample was run on SDS-PAGE gel and transferred to the PVDF membrane. After blocking the membrane at room temperature with 5% milk, the membranes were probed with Anti-Nrf2 (ab62352, Abcam, Waltham, MA, USA) and Anti-Prdx6 antibodies (Ab Frontier, Seoul, Republic of Korea). Tubulin (ab44928, Abcam, Waltham, MA, USA) and LaminB1 (ab133741, Abcam) were used as internal control. Following incubation with the primary antibody, PVDF membranes were incubated with secondary antibody (sc-2357 and sc-516102, Santa Cruz Biotechnology, Dallas, TX, USA), and protein bands on membrane were visualized by incubating the membrane with luminol reagent (sc-2048; Santa Cruz Biotechnology, Dallas, TX, USA). Protein band images were recorded with a FUJIFILM-LAS-4000 luminescent image analyzer (FUJIFILM Medical Systems Inc., Hanover Park, IL, USA).

### 2.9. Quantitative Real-Time PCR

The relative levels of Nrf2 and its target genes mRNA were measured by quantitative real-time PCR (RT-qPCR). Total RNA was isolated from Hyd-treated SRA-hLECs as well as mLECs/lenses derived from C57BL/6 mice by using the single-step guanidine thiocyanate/phenol/chloroform extraction method (Trizol Reagent, Invitrogen, Waltham, MA, USA), as described previously [2,19,31]. cDNA synthesis was conducted with 5 µg of total RNA using Superscript II RNAase H-reverse-transcriptase. RT-qPCR reactions were prepared using SYBR Green Master Mix (Roche Diagnostic Corporation, Indianapolis, IN, USA), specific primers (as shown in Table 1), and with diluted cDNA in a Roche^®^ LC480 Sequence detector system (Roche Diagnostic Corporation, Indianapolis, IN, USA). RT-qPCR was performed using program condition of 5 min hot start at 95 °C, followed by 45 cycles for 10 s (seconds) at 95 °C, 30 s at 60 °C, and 10 s at 72 °C. Data were analyzed by LightCycler480 software by Roche. All data analysis was performed in triplicate. The relative values of target genes were normalized to the control using corresponding β-actin as an internal control.

### 2.10. Assessment of Phospholipase A_2_ (PLA_2_) Activity

Phospholipase A_2_ (PLA_2_) activity was carried out in accordance with the manufacturer’s protocol (EnzChek Phospholipase A2 kit; E10217, Invitrogen, Waltham, MA, USA) and our published protocol [3,81,83]. Briefly, SRA-hLECs and mLECs were treated with different concentrations of Hyd for 24 h. Thereafter, total protein was isolated and quantified by BCA protein assay (ThermoFisher Scientific, Waltham, MA, USA). To prepare a standard curve, different concentrations (0–10 Units/mL) of PLA_2_ was made by diluting PLA_2_ stock solution (500 Units/mL) with 1 × reaction buffer up to 50 µL. For sample preparation, an equal amount of protein was diluted with 1 × PLA_2_ reaction buffer to make volume up to 50 µL. The reaction was started by adding 50 µL of the substrate-liposome mix to each microplate well consisting of control, standard, and the samples with 100 µL total reaction volume. The fluorescence units were measured at optical density (O.D.), Ex485 nm/Em535 nm, using a microplate reader (DTX 880, Multimode Detector, Molecular device, San Jose, CA, USA).

### 2.11. Estimation of Glutathione (GSH) Peroxidase Activity

Glutathione (GSH) peroxidase activity was quantified according to company’s protocol (Glutathione Peroxidase activity kit, Cat No. ADI-900-158, Enzo Life Sciences, Farmingdale, NY, USA) and our published protocol [3,81,83]. Total cell lysate was prepared from Hyd-treated or untreated SRA-hLECs or mLECs. The equal amount of quantified protein was subjected to quantify the enzymatic activity. To set up the reaction, 20 µL of standard (glutathione peroxidase) or samples or control, and 140 µL of 1 × assay buffer, with 20 µL of 10× reaction mix were added to each well of a 96-well plate. An amount of 20 µL of cumene hydroperoxide was quickly added to each well to initiate the reaction. Thereafter, O.D. was measured at absorbance 340 nm every 1 min up to 15 min period. Blank O.D. was subtracted from the standard as well as sample O.D. to obtain the net rate of absorbance at 340 nm for the GSH peroxidase activity (DTX 880, Multimode Detector, Molecular device, San Jose, CA, USA).

### 2.12. Quantitation of Superoxide Dismutase (SOD) Activity

Superoxide Dismutase (SOD) enzymatic activity was measured as mentioned in the manufacturer’s protocol (Superoxide Dismutase (SOD) Assay, Kit, LS-K224, LifeSpan BioSciences, Inc., Seattle, WA, USA) [3]. To prepare a standard curve, SOD enzyme was diluted using diluent at different concentrations (0–3 U/mL). Standards were prepared and 20 µL was added to a 96-well microplate. For samples, an equal amount of Hyd-treated or untreated SRA-hLECs and mLECs cellular protein and dilutions were added to separate wells of a 96-well plate with 20 µL volume. An amount of 160 µL of working reagent (mixture of 160 µL assay buffer, 5 µL xanthine, and 5 µL WST-1) was added to the standard, samples, and control wells. The contents were mixed by tapping the plate, 20 µL of diluted XO enzyme (1:20 diluted in diluent) was added, and immediately the O.D. was measured at 440 nm and 0 min (OD_0_) with DTX 880 Multimode Detector (SpectraMAX Gemini, San Jose, CA, USA). The plate was incubated at room temperature in the dark for 60 min, and, again, the O.D. was measured at 440 nm (OD_60_). Each standard and sample well were calculated using the following formula, ΔOD_60_ = OD_60_ − OD_0_, and was presented in percentage.

### 2.13. Quantitation of Catalase Activity

Catalase activity was determined following the manufacturer’s instructions (Catalase Assay, Kit, LS-K245, LifeSpan BioSciences, Inc., Seattle, WA, USA) [3]. To prepare a catalase standard curve, 10 µL of different concentrations of H_2_O_2_ (0, 120 µM, 240 µM and 400 µM) of each standard and 90 µL of assay buffer were added into separate wells of a 96-well plate and proceeded for detection. For samples, 10 µL of an equal amount of total protein isolated from Hyd-treated SRA-hLECs and mLECs were separately added into wells of a 96-well plate. To initiate the catalase reaction, 90 µL of freshly prepared 50 µM H_2_O_2_ substrate (1 µL of the 4.8 mM H_2_O_2_ with 95 µL assay buffer) was added to the blank, control, and sample wells. A 96-well plate was incubated for 30 min at room temperature. After quick mix, the plates were submitted for detection. For standard, samples, and controls detection, 100 µL of detection reagent (mixture of 102 µL assay buffer, 1 µL eye reagent, 1 µL HRP enzymes) was added to each reaction well of standard, samples, and controls. After 10 min incubation, optical density (O.D.) was measured at 570 nm with DTX 880 Multimode Detector (SpectraMAX Gemini, San Jose, CA, USA).

### 2.14. Isolation of Cytosol and Nuclear Protein Fractions

Cytosolic or nuclear fraction was isolated following our previously published methods [3,10,19,84]. Briefly, SRA-hLECs or mLECs were cultured in 100-mm plates overnight. Next-day cells were treated with Hyd for 24 h. For cytosolic fraction, cells were washed thrice with chilled phosphate-buffered saline (pH 7.4) and collected by centrifugation. The pellet obtained was suspended in 5 pellet volumes of cytoplasmic extraction buffer (10 mM HEPES (adjusted pH at 7.9), 0.1 mM EDTA, 10 mM KCl, 0.4% (*v*/*v*) Nonidet P-40, 1 mM DTT, 0.5 mM phenylmethylsulfonyl fluoride (PMSF) and protease inhibitor). After a brief incubation on ice, it was centrifuged at 10,000 rpm for 10 min. Supernatant comprising the cytosolic fraction was transferred to a fresh tube. The pellet obtained was lysed in nuclear extract buffer (20 mM HEPES (adjusted pH at 7.9), 1 mM EDTA, 0.4 M NaCl, 10% (*v*/*v*) glycerol, 1 mM DTT, 0.5 mM PMSF and protease inhibitor) and subjected to continuous vortexing at 4 °C for 2 h to obtain the nuclear protein fraction. Finally, the extract was spun at 14,000 rpm for 15 min to pellet the nuclei. After centrifugation, the nuclear extract was aliquoted, and aliquots were stored at −80 °C. Protein was estimated according to the Bradford protein assay method, and the extract was used for the present study.

### 2.15. Determination of Nrf2-DNA (ARE) Binding Activity

The nuclear fraction was isolated, as noted above in Section 2.14. Nrf2-DNA binding activity was measured using kit TransAM Nrf2 Transcription Factor Assay Kit (Cat. No. 50296, Active motif, Carlsbad, CA, USA) and as described earlier [2,10,31]. Nuclear extract (10 µg) from Hyd-treated or untreated SRA-hLECs and mLECs were added to a well coated with oligonucleotides containing ARE (antioxidant response element) sequences, or it is mutated at ARE consensus binding sequences to each sample well. For blanks, 10 µL of complete lysis buffer was used. The controls and samples wells were incubated at room temperature (RT) for 1 h. The 100 µL primary antibody (1:1000 in binding buffer) was added after washing the wells and incubated at RT for 1h. After three washings, 100 µL anti-rabbit HRP conjugated antibody (1:1000 dilution) was added and further incubated for 1h at RT. By the addition of 100 µL of developing solution to the wells, the reaction was developed. The reaction was stopped by adding 100 µL of stop solution, and a reading was taken at O.D. 450 nm using a microplate reader (DTX 880, Multimode Detector, Molecular device, San Jose, CA, USA).

### 2.16. Chromatin Immunoprecipitation (ChIP) Assay (In Vivo DNA Binding Assay)

ChIP analysis was conducted by using the ChIP-IT^®^ Express kit (Cat. No. 53008; Active Motif, Carlsbad, CA, USA) and ChIP-IT^®^ qPCR analysis kit (Cat. No. 53029; Active Motif, Carlsbad, CA, USA) following manufacturer’s protocol and as described previously [2,3,19,80,85]. Briefly, cells were processed following the protocol, and the fixation reactions were halted by addition of Glycine Fix-Stop solution. Cells were collected, and the cell pellet was disrupted in 1 mL ice-cold lysis buffer using a Dounce homogenizer. Released nuclei were resuspended in shearing buffer after centrifugation. Chromatin was then disrupted to obtain 200–300 bp using an ultrasonic cell disruptor (Microson, Farmingdale, NY, USA). Chromatin samples were used to assess DNA and transcription factor(s) enrichment by using control IgG, antibody specific to Nrf2 (ab62352, Abcam), to pull down the specific DNA fragment and then followed by RT-qPCR reaction. RT-qPCR was performed at 2 min at 95 °C, 15 s at 95 °C, 20 s at 58 °C, and 20 s at 72 °C for 40 cycles in 20 μL reaction volume (RT-qPCR). The results derived from RT-qPCR are presented as histograms.

### 2.17. Preparation of Prdx6 Promoter-Fused to Chloramphenicol Acetyltransferase (CAT) Reporter Vector

Human genomic DNA was used to construct pCAT-hPrdx6 promoter plasmid, as reported previously in our published protocol [2,10,31]. In brief, isolated genomic DNA was subjected to genomic-PCR with region specific primers, and the 5′-flanking regions, ranging from −918 to +30 bp, were isolated by using Advantage^®^ Genomic PCR Kit (Cat. No. 639103 and 639104, Clontech Laboratories, Inc., Mountain View, CA, USA). The PCR product obtained was amplified and verified by sequencing as described previously [19,74]. To engineer pCAT-linked hPrdx6 promoter plasmid, the DNA fragment (−918 to +30 bp) was ligated into basic pCAT vector (Promega, Madison, WI, USA) at the *SacI* and *XhoI* sites. Primers used were as follows: forward primer 5′-GACAGAGTTGAGCTCCACACAG-3′ and reverse primer 5′-CACGTCCTCGAGAAGCAGAC-3′. Expression and purification of recombinant protein TAT-HA-Prdx6 was prepared as described in our published report [83,86]. Hyd and N-acetyl-L- cysteine (NAC) were purchased from Sigma (Catalog No. A9165, Millipore Sigma, Rockville, MD, USA).

### 2.18. Nrf2 Knock down Experiment

To generate Nrf2-depleted SRA-hLECs, Nrf2 was knocked down using human Nrf2 specific *sh*RNA (*Sh*-Nrf2, sc-37030-SH, SantaCruz Biotechnology, Dallas, TX, USA) plasmid. A scrambled *sh*RNA was used as negative control (*Sh*-Control, sc-108060, SantaCruz Biotechnology, Dallas, TX, USA). SRA-hLECs were transfected with control *sh*RNA or Nrf2 *sh*RNA by using Neon^TM^ Transfection System. After 2 days of transfection, transfectants were used for the study or selected with selection marker puromycin to make stable cell lines. SRA-hLECs were transfected by using the Neon Transfection System (Invitrogen, Waltham, MA, USA) as described in our published protocol [10,19,31,81].

### 2.19. Lens Organ Culture and H_2_O_2_ Treatment

Hyd (left eye) or buffered saline (7.2) (right eye) was topically instilled in 16 or 22-M-old mice eyes for 7 or 15 days once daily (please see illustration in Results Section). At day 8 or 16, lenses were carefully isolated and were cultured in 48-well culture plates containing 199 media without any supplements. After 24 h, the clear lenses were subjected to H_2_O_2_-induced oxidative stress. The lenses were observed routinely. After 48 h, oxidative stress lenses were photographed using Nikon SMZ 745T microscope fitted with a Nikon camera and a computer loaded with an analysis software program [51,54,83,87]. To examine the effects of Hyd, the lenses were processed to measure levels of ROS, lipid peroxidation, protein carbonyls and oxidative DNA damage, and Nrf2 and antioxidant genes, as described above and in the Results and Legends Sections.

### 2.20. Statistical Analysis

All experiments were performed with at least three independent biological replicas. Results are expressed as the average mean ± standard deviation (S.D.). Statistical analyses were performed by Sigma plot. For post-hoc statistical tests, we performed Bonferroni and Holm–Sidak test using one-way ANOVA, these tests can be used for both comparisons versus a control group (compare several treatment groups to a single control group) and pairwise comparisons (comparison of the mean responses to the different treatment groups). Statistical significance between control and treatment groups was assessed, and *p* value < 0.05 and <0.001 was considered statistically significant.

## 3. Results

### 3.1. Hyd Efficaciously Rescued Lens Epithelial Cells under Oxidative Stress

**Figure 1:**
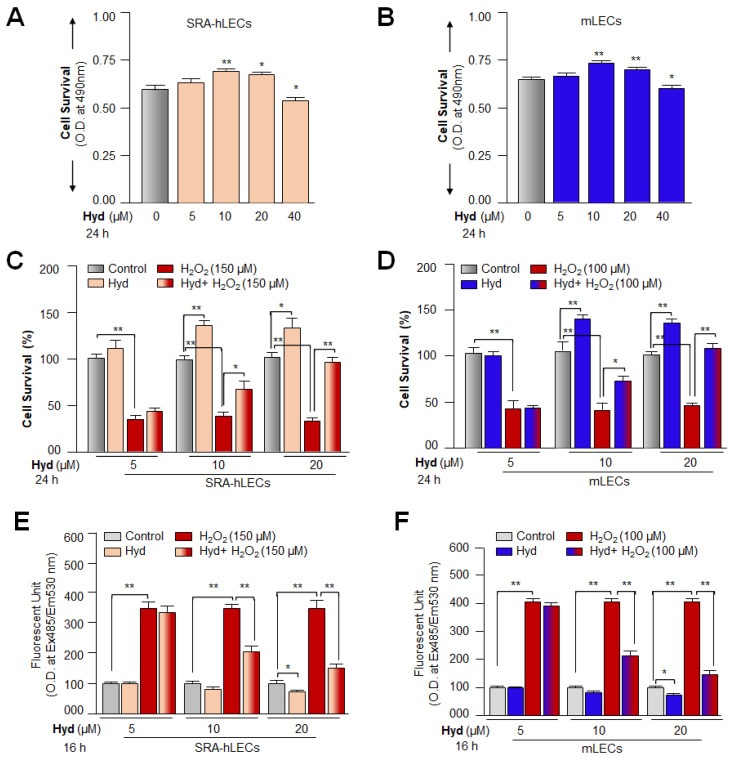
Hyd protects LECs from H_2_O_2_-induced oxidative cell death. (**A**,**B**) Viability assay showing the dose-dependent effect(s) of Hyd on survival of SRA-hLECs and mLECs. Cultured SRA-hLECs and mLECs were treated with different concentrations of Hyd to determine its nontoxic concentration using MTS assay. The data represent the mean ± S.D. of three independent experiments. Untreated vs. Hyd-treated LECs; * *p* < 0.05; ** *p* < 0.001. (**C**,**D**) The cell viability of LECs under H_2_O_2_-induced oxidative stress was significantly improved with Hyd treatment. Cultured SRA-hLECs and mLECs were exposed to H_2_O_2_ in absence or presence of Hyd for 24 h as indicated in Figure 1, and cell viability was performed using MTS assay. Histograms represent the mean ± S.D. value of three independent experiments. * *p* < 0.05; ** *p* < 0.001. (**E**,**F**) Hyd defends LECs against H_2_O_2_-induced cell death by reducing the ROS levels. Hyd-treated and untreated SRA-hLECs and mLECs were exposed to H_2_O_2_. Then, 16 h later, ROS levels were quantified with H_2_-DCF-DA dye method. Histogram represents the mean ± S.D. of three independent experiments. * *p* < 0.05; ** *p* < 0.001.

In addition to antihypertensive property, Hyd has been shown to exert its cytoprotective and antiaging effects by activating the Nrf2/ARE antioxidant pathway in various cell types [21,22,88,89]. Therefore, at first, we confirmed whether Hyd protects LECs against oxidative stress as reported previously for other cell types. As shown in Figure 1A, we treated LECs with different concentrations of Hyd for 24 h. We found that 20 µM of Hyd was nontoxic, and treated LECs survived better with an increase in cell growth. Interestingly, a quantitation of ROS using H_2_-DCF-DA dye revealed that Hyd treatment itself does not augment intracellular ROS levels in the cells, but it reduces the levels of ROS when examined after 8 h of treatment. The results indicated that Hyd does not act as a pro-oxidant. Next, to determine the functional significance of Hyd treatment, we assessed the survival levels of Hyd-treated or untreated LECs (SRA-hLECs or mLECs) using MTS assay against oxidative stress, as shown in Figure 1C–F. As expected, Hyd-treated LECs conferred resistance against H_2_O_2_-induced oxidative stress. Furthermore, assessment by H_2_-DCF-DA dye showed a significant reduction of ROS in these treated cells compared to untreated ones, suggesting that Hyd protected the LECs by reducing intracellular ROS. Our experiments also indicated that 20 µM could be a more effective concentration in defending LECs from oxidative damage. It is worth noting that LECs treated with Hyd displayed a significant reduction in the ROS levels of the cells untreated with H_2_O_2,_ indicating that Hyd is not a pro-oxidant. Based on these observations, we surmised that Hyd might provide its protective effect by activating the antioxidant pathway in LECs as reported previously in other model systems. Thus, we focused our present study to understand Hyd-mediated molecular event(s), which might be involved in the activation of Nrf2/ARE antioxidant pathway and cellular protection.

### 3.2. Hyd Efficaciously Blunted Oxidative Stress-Driven Aberrant Cellular Levels of Lipid Peroxidation and Rescued Oxidative Protein and DNA

**Figure 2:**
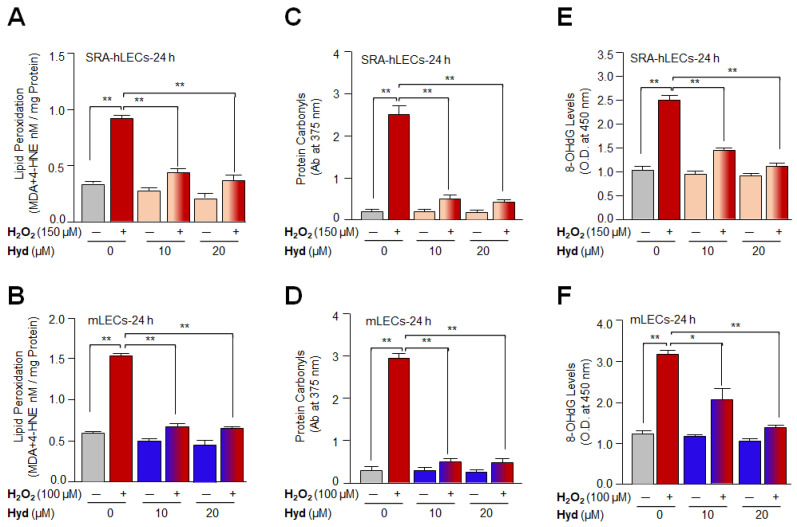
Hyd treatment attenuated H_2_O_2_-induced accelerated production of lipid peroxidation, protein carbonyls, and DNA damage. (**A**,**B**) Hyd blunted lipid peroxidation in H_2_O_2_-exposed LECs. Hyd-treated or untreated LECs were exposed to H_2_O_2_ and then processed for LPO assay. A significant reduction of MDA + 4-HAE levels were observed in Hyd-treated LECs exposed to H_2_O_2_ compared to untreated control. Histogram represents the mean ± S.D. of three independent experiments. ** *p* < 0.001. (**C**,**D**) Protein carbonyls were measured using 2-DNPH assay in LECs treated with Hyd or H_2_O_2_ or Hyd plus H_2_O_2_. Hyd treatment significantly lowered the protein carbonyl levels raised by H_2_O_2_ exposure. Histogram represents the mean ± S.D. of three independent experiments. ** *p* < 0.001. (**E**,**F**) Hyd treatment blunted oxidative stress-induced DNA damage in LECs. LECs exposed to H_2_O_2_ alone or cotreated with Hyd for 24 h were subjected to measure the 8-OHdG levels. Data showed that Hyd attenuated oxidative DNA damage in LECs induced by H_2_O_2_ exposure. Histogram represents the mean ± S.D. of three independent experiments. * *p* < 0.05; ** *p* < 0.001.

After confirming that Hyd protects LECs by mitigating ROS accumulation and considering the impact of oxidative-induced generation of toxic products-mediated aberrant modifications of proteins and DNA damage, we tested whether the Hyd halts oxidative DNA damage or attenuates the levels of oxidative stress-induced formation of toxic adducts, such as MDA (malondialdehyde)/4-HNE (4-hydroxyalkenals) or protein carbonylation. To achieve this end, we exposed Hyd-pretreated or untreated LECs to H_2_O_2_ for 24 h, and the levels of MDA+4-HNE, protein carbonyls, as well as DNA damage, were quantified. We observed that H_2_O_2_-treated LECs displayed a significant increase in MDA plus 4-HNE levels (Figure 2A,B; gray vs. red bar), which were significantly reduced by Hyd treatment (Figure 2A; SRA-hLECs, red vs. light orange-red bars and Figure 2B; mLECs, red vs. blue-red bars). Next, we quantified levels of carbonyl groups in Hyd-treated and untreated LECs using a 2,4-DNPH (dinitrophenylhydrazine) assay. We found that H_2_O_2_-induced increase in protein carbonyl content (Figure 2C,D; gray vs. red bar) could be significantly reduced in Hyd-treated LECs (Figure 2C; SRA-hLECs, red vs. light orange-red bars and Figure 2D; mLECs, red vs. blue-red bars). Furthermore, the formation of 8-OHdG is a ubiquitous marker of oxidative stress among various types of oxidative DNA damage. We, therefore, examined whether Hyd can reduce the levels of 8-OHdG formation in LECs facing oxidative stress. To this end, we found that LECs treated with H_2_O_2_ alone displayed an increased amount of 8-OHdG (Figure 2E,F; gray vs. red bar) compared to Hyd-treated LECs, showing a significant reduction in the levels of 8-OHdG (Figure 2E; SRA-hLECs, red vs. light orange-red bars and Figure 2F; mLECs, red vs. blue-red bars). Thus, the results revealed that Hyd has the ability to protect DNA from oxidative stress.

### 3.3. Hyd Activated the Nrf2/ARE-Driven Luciferase Activity in LECs

**Figure 3:**
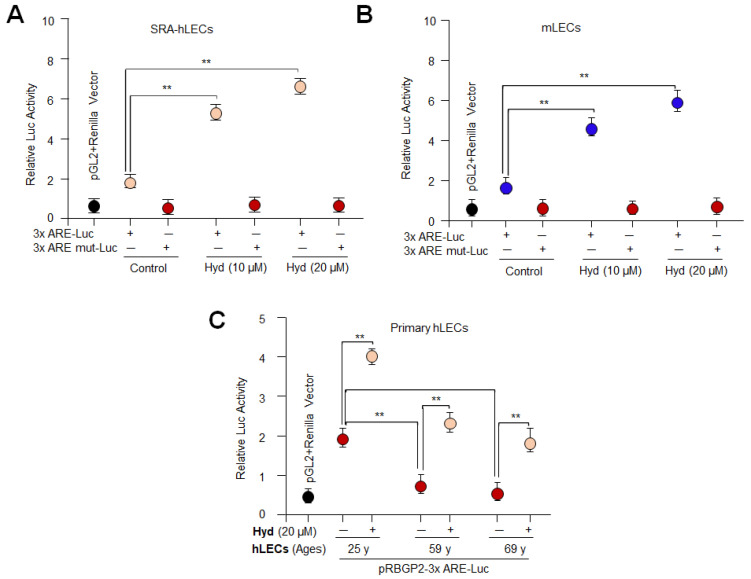
Hyd-activated antioxidant gene transcription via Nrf2/ARE in general; Engineered luciferase promoter containing 3x ARE sites suggested that Hyd activated the Nrf2-dependent transcription through ARE sequences in LECs. (**A**,**B**) SRA-hLECs (**A**) and mLECs (**B**) were transiently transfected with pRBGP2-3xARE-LUC plasmid or its mutant at all three ARE sites. Then, 14 h later, the transfected LECs were treated with different concentrations of Hyd for 24 h, as indicated. Relative LUC activity was observed. The data represent the mean ± S.D. from three independent experiments. ** *p* < 0.001. (**C**) Progressive decline in Nrf2/ARE-mediated transcription with advancing age was reactivated by Hyd treatment. Primary hLECs of different age groups were transiently transfected with pRBGP2-3xARE-LUC plasmid. At 14 h of post-transfection, hLECs were treated with Hyd (20 µM) for 24 h as indicated, and relative LUC activity was quantified. The data represent the mean ± S.D. values obtained from three independent experiments. ** *p* < 0.001.

Previously, we have reported that Nrf2/ARE activation-dependent increased antioxidant gene transcription is directly related to expression and activities of antioxidants [2,10,19,21,31]. Given the results of Figure 1 showing the reduction of ROS levels in Hyd-treated cells, we surmised that Hyd’s cytoprotective property should be attributed to activation of Nrf2/ARE transcription. To determine whether Hyd activates the Nrf2/ARE pathway, we performed Hyd-dependent Nrf2/ARE transactivation experiments. Toward this, we made use of pRBGP2-3x-ARE-LUC containing three ARE sites or its corresponding mutants pRBGP4-3xmut ARE-LUC (a kind gift from Hozumi Motohashi, Japan) [82]. As shown in Figure 3, SRA-hLECs or mLECs or different ages of primary hLECs were transfected with pRBGP2-3x-ARE-LUC or pRBGP4-3xmut ARE-LUC along with *Renilla*, pRL-TK vector (Promega, Madison, WI, USA). Transfectants treated with different concentrations of Hyd for 24 h were assayed for luciferase activity using Dual-Glo luciferase assay system (Promega, Madison, WI, USA). Data analysis disclosed that Hyd significantly up-regulated the Nrf2/ARE-mediated transcription in a dose-dependent manner, as shown in Figure 3A,B. However, in aging hLECs, 20 µM of Hyd could significantly reactivate the Nrf2/ARE-driven transcription under the same experimental conditions as shown in Figure 3C, suggesting that aging LECs required higher concentration of Hyd to reinforce Nrf2/ARE-mediated transcription. The results emphasized that the Nrf2/ARE-mediated signaling pathway can be reactivated by Hyd in aging LECs. Additionally, the results opened the door to design further experiments to define Hyd-mediated molecular mechanisms involved in the regulation of the antioxidant genes bearing ARE sites, such as Prdx6.

### 3.4. Hyd Amplified the Antioxidants Expression via Activating Nrf2/ARE Pathways in LECs

**Figure 4:**
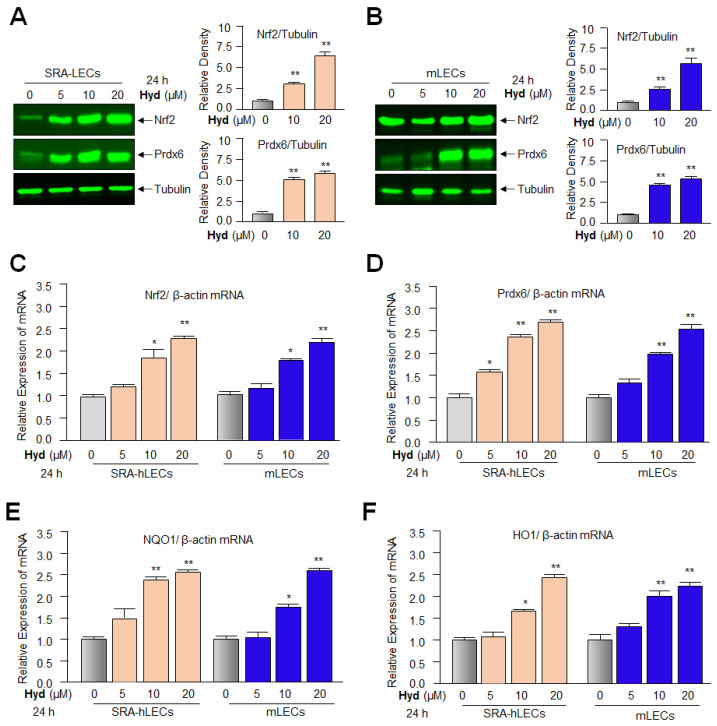

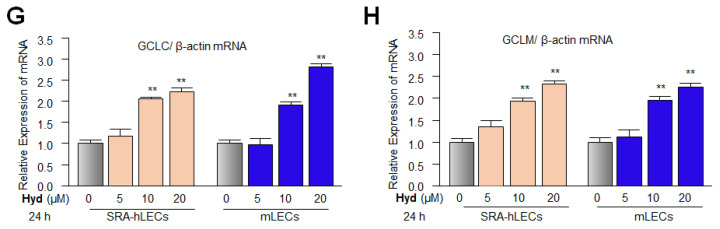
Hyd efficiently activated the Nrf2 antioxidant signaling pathway in LECs. (**A**,**B**) Hyd increased the cellular abundance of Nrf2 and Prdx6 protein in a dose-dependent fashion, as demonstrated by Western analysis of total cell lysate. Total cell lysate was isolated from SRA-hLECs (**A**) and mLECs (**B**) treated with different concentrations of Hyd for 24 h. Nrf2 and Prdx6 protein expression were analyzed using their corresponding specific antibodies; tubulin served as internal control. Nrf2 and Prdx6 protein band were scanned and quantified using a densitometry and were normalized with corresponding tubulin levels. Histograms represent the relative density values that were depicted on the right side of the protein band. The data represent the mean ± S.D. values of three independent experiments. Untreated vs. Hyd-treated LECs; ** *p* < 0.001. (**C**–**H**) Hyd significantly augmented levels of Nrf2 and its major target antioxidant genes examined, such as Prdx6, NQO1, HO1, GCLC, and GCLM. LECs were treated with different concentrations of Hyd for 24 h. Total RNA were isolated and processed for real-time PCR using specific primers. Data represent mean ± S.D. values of three independent experiments. Untreated vs. Hyd-treated LECs; * *p* < 0.05; ** *p* < 0.001.

The results derived from the above experiments implied that Hyd can reactivate Nrf2/ARE-mediated transcription in general. This argues that Hyd may exert its protective function by up-regulating Nrf2/antioxidant expression in LECs. To examine this, we used multiple LECs types, primary hLECs, SRA-hLECs, and mLECs. At first, we quantified Nrf2 and its target gene, Prdx6, at protein levels in Hyd treated/untreated LECs. Immunoblot analysis revealed that the cellular extracts of LECs treated with different concentrations of Hyd (0, 5, 10, and 20 µM for 24 h) had a concentration-dependent effect(s) on the increase in Nrf2 and Prdx6 protein expression (Figure 4A,B). However, unexpectedly, 5 µM of Hyd could not induce Nrf2 and Prdx6 expression in mLECs as observed in hLECs, suggesting that mLECs may be less responsive due to their limited permeability to Hyd, or Hyd may have cell/tissue specificity. Furthermore, because Nrf2 up-regulates antioxidant genes as well as its own expression [90] through remodeling of transcriptional machinery, we next examined the relative expression levels of mRNA (transcripts) of Nrf2 (Figure 4C) and its major target antioxidant genes such as Prdx6, NQO1, HO1, GCLC, and GCLM by RT-qPCR in SRA-hLECs and mLECs treated with Hyd (Figure 4D–H). Quantitation of these genes by RT-qPCR demonstrated a dose-dependent increase in Nrf2 and its downstream target genes at 10 and 20 µM of Hyd.

### 3.5. Hyd Treatment Enhanced the Enzymatic Activities of Antioxidant Genes

**Figure 5:**
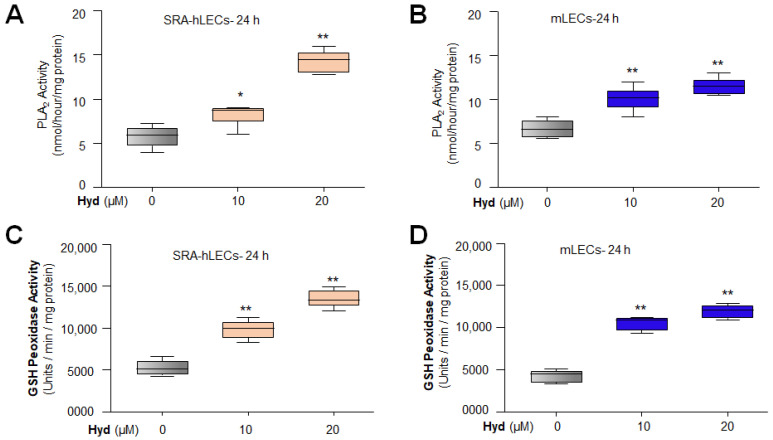

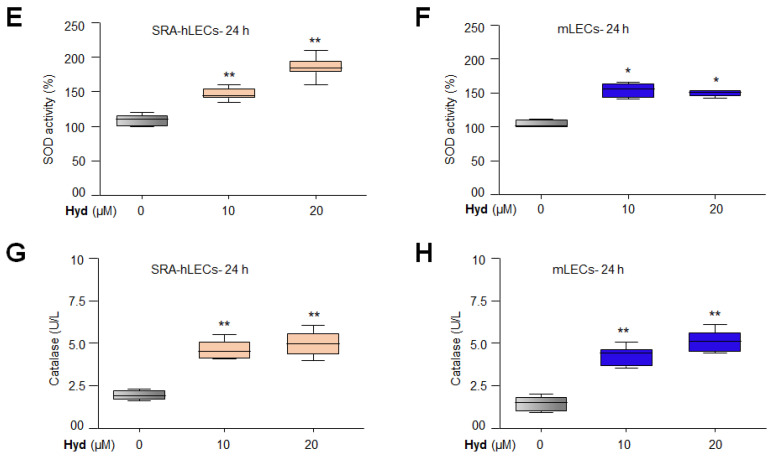
Hyd treatment increased the enzymatic activities of antioxidant genes significantly. Total cell lysate isolated from Hyd-treated or untreated SRA-hLECs (**A**,**C**,**E**,**G**) and mLECs (**B**,**D**,**F**,**H**) for 24 h were used to analyze the enzymatic activities as indicated. The same amount of protein was processed to measure PLA_2_ ((**A**) SRA-hLECs; (**B**) mLECs), GSH peroxidase ((**C**) SRA-hLECs; (**D**) mLECs), SOD ((**E**) SRA-hLECs; (**F**) mLECs), and catalase ((**G**) SRA-hLECs; (**H**) mLECs) activities following the company’s protocols. The data represent the mean ± S.D. values of three independent experiments. Untreated vs. Hyd-treated LECs; * *p* < 0.05; ** *p* < 0.001.

Considering our previous report that antioxidant genes expression and activities were deteriorated during aging and that expression or activity can be reactivated by Metformin [3], we are, therefore, inclined to know whether, similar to Metformin, Hyd can amplify the enzymatic activities of antioxidants, such as phospholipase A_2_ (PLA_2_; Figure 5A, SRA-hLECs; Figure 5B, mLECs), glutathione (GSH) peroxidase (Figure 5C, SRA-hLECs; Figure 5D, mLECs), superoxide dismutase (SOD; Figure 5E, SRA-hLECs; Figure 5F, mLECs), and catalase (Figure 5G, SRA-hLECs; Figure 5H, mLECs) enzymatic activities. Figure 5 shows a significant increase in enzymatic activities of all antioxidants examined in Hyd-treated LECs. However, we anticipate that Hyd may alter the antioxidants’ conformation, resulting in an increase in antioxidant enzymatic activities. Taken together, our data indicated that Hyd treatment significantly increased the Nrf2’s target antioxidant gene’s expression as well as their enzymatic activities, as shown in Figure 5.

### 3.6. Hyd Triggered the Nuclear Accumulation of Nrf2 and Augmented the Nrf2 Activity in Concentration-Dependent Fashion

**Figure 6:**
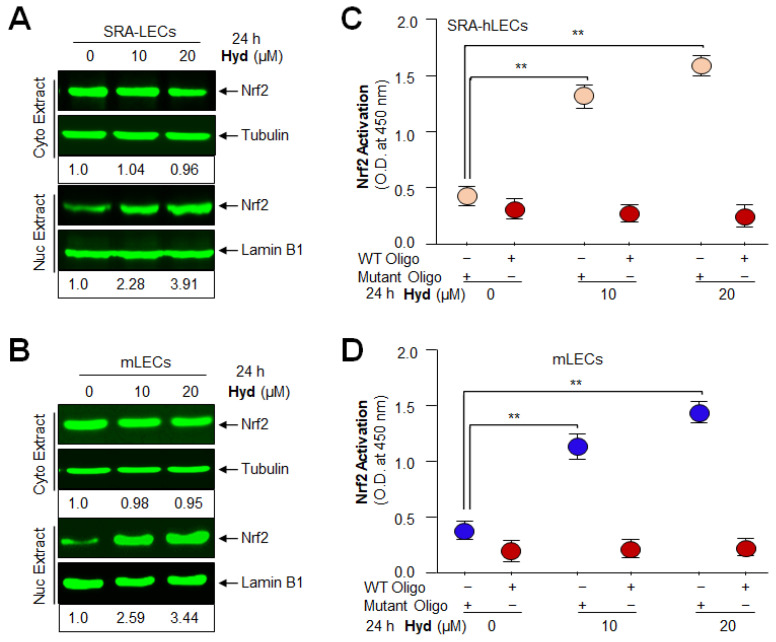
Hyd augmented the nuclear accumulation of Nrf2 and promoted the Nrf2/ARE activity in a dose-dependent manner. (**A**,**B**) Hyd induced increased nuclear accumulation of Nrf2 in LECs. Cultured SRA-hLECs (**A**) and mLECs (**B**) were treated with different concentrations of Hyd for 24 h. Cytosol and nuclear protein extract were immunoblotted with antibody specific to Nrf2. Tubulin and lamin B1 were used as loading control. Numbers under each protein band demonstrate densitometry value. (**C**,**D**) Hyd-treated LECs displayed significantly enhanced Nrf2 activity. Nuclear extracts from SRA-hLECs (**C**) and mLECs (**D**) treated with different amounts of Hyd for 24 h were analyzed for Nrf2/ARE interaction by ELISA (enzyme-linked immunosorbent assay). An equal amount of nuclear protein was processed and assayed for Nrf2 activity using a commercially available kit (Active motif). The data represent the mean ± S.D values from three independent experiments. *p* values were determined Hyd-treated vs. untreated control. ** *p* < 0.001.

Because nuclear accumulation of Nrf2 is a prerequisite for its transcriptional activity, we next pursued determining the effect of Hyd on the cellular status of Nrf2. To this end, LECs were treated with Hyd for 24 h. Immunoblotting of nuclear and cytosolic fractions with antibody specific to Nrf2 showed that nuclear accumulation of Nrf2 was increased in Hyd-treated LECs, and accumulation was increased at 20 μM of Hyd (Figure 6A,B, lower panel). However, unexpectedly, we observed a very small change (not significant change) in the abundance of Nrf2 in the cytosolic fraction of LECs. Having observed that Hyd facilitates Nrf2’s nuclear accumulation, next we asked whether Hyd-induced increased enrichment of Nrf2 is functionally active. To examine this, we performed the Nrf2 transactivation assay. Equal amounts of nuclear fraction isolated from SRA-hLECs (Figure 6C) and mLECs (Figure 6D) were used to assess the Nrf2’s activity by using Trans-Nrf2 transcription factor assay kit (Active Motif). Data of Figure 6C,D demonstrated that the DNA binding activity of the Nrf2 was increased with the increase in Hyd concentration, suggesting that accumulated Nrf2 in the nucleus was functionally active, and Hyd may act via activating the Nrf2/ARE pathway by enhancing Nrf2’ s nuclear accumulation.

### 3.7. In Vivo DNA Binding and Transactivation Assays Disclosed That Hyd Activated Antioxidant Transcription via Nrf2/ARE Mechanism

**Figure 7:**
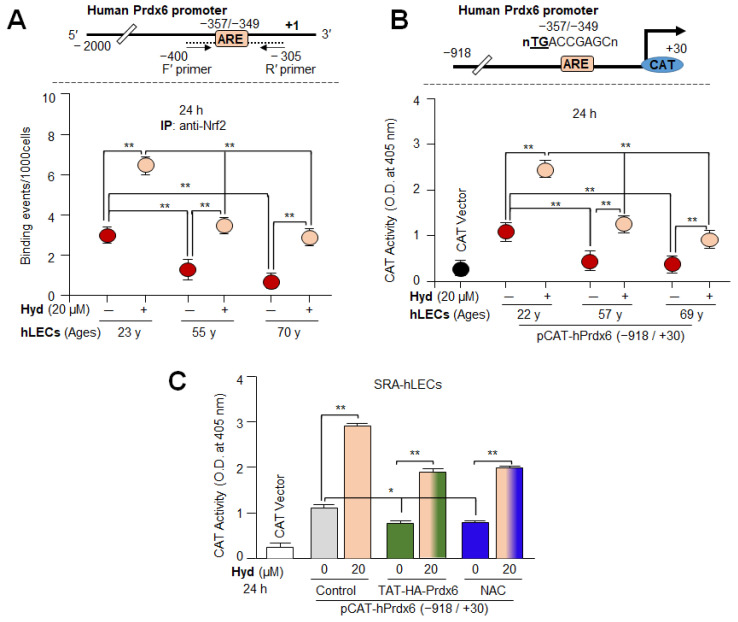
(**A**) In vivo DNA binding assay disclosed that Hyd reinforced binding activity of Nrf2 in aging hLECs. Upper panel, schematic illustration of Prdx6 gene promoter with ARE site and ChIP primer locations. Lower panel, in vivo DNA-binding (ChIP) assay revealed that Hyd reinforced the binding activity of Nrf2 to its ARE sequences of the *Prdx6* promoter in aging hLECs. ChIP assay was conducted using ChIP grade antibody specific to Nrf2. Immunoprecipitated DNA fragments isolated from vehicle control or Hyd-treated different ages of primary hLECs were purified and processed for ChIP-RT-qPCR analysis. The reduction in Nrf2 enrichment at ARE site in untreated LECs, while increased abundance of Nrf2 at the ARE site in response to Hyd treatment, could be significantly eminent. Young (23 y) vs. aged (55 y and 70 y) subjects and untreated vs. Hyd treated, ** *p* < 0.001. (**B**) Age-related reduction in transcriptional activity of *Prdx6* promoter in hLECs of variable ages was restored by Hyd. Top panel, diagrammatic sketch of the 5′- constructs of hPrdx6 promoter (−918/+30 bps) linked to CAT reporter plasmid. Lower panel, CAT activity of *Prdx6* promoter and CAT vector. The data represent the mean ± S.D. from three independent experiments. Younger age (22 y) vs. aging (57 y and 69 y) samples; untreated vs. Hyd-treated hLECs; ** *p* < 0.001. (**C**) Hyd-mediated Nrf2 activation of Prdx6 gene promoter was ROS independent. Hyd treatment significantly increased the *Prdx6* promoter activity in a dose-dependent manner. The ARE-driven *Prdx6* promoter activity was decreased in SRA-hLECs treated with TAT-HA-Prdx6 (10 µg/mL) protein or antioxidant molecules N-acetyl cysteine (NAC, 5 *m*M) alone for 24 h. At the same time, Prdx6 transcription increased in SRA-hLECs when cotreated with TAT-HA-Prdx6 protein or NAC and Hyd. The data represent the mean ± S.D. from three independent experiments. * *p* < 0.05; ** *p* < 0.001.

To test the ability of Hyd in activating/reactivating Nrf2/ARE binding in aging/aged hLECs, we performed the chromatin immunoprecipitation (ChIP) assay to examine the enrichment of Nrf2 on ARE sequences of the hPrdx6 gene promoter. Primary hLECs of variable ages treated with Hyd for 24 h were subjected to ChIP assay with antibody specific to Nrf2. As shown in Figure 7A, the Nrf2 enrichment at ARE sequences in *Prdx6* promoter was significantly increased in Hyd-treated, aging primary hLECs. However, younger hLECs were relatively more responsive to Hyd treatment compared to aging hLECs. This indicated that the aging hLECs retained Nrf2 activity and can be reactivated by means of Hyd treatment. Furthermore, to examine whether Hyd-induced increased Nrf2 binding to the *Prdx6* promoter was functional, we carried out transcription assay with the *Prdx6* promoter-linked to CAT reporter plasmid by transfecting hLECs. We found that Hyd could significantly amplify the promoter activity from its basal activity levels in all aging cells (Figure 7B). As expected, younger cells were more responsive than the aged hLECs as observed in the Nrf2/ARE binding experiments (Figure 7A). Taken together, our in vivo DNA binding assay coupled with the transactivation assay demonstrated that Hyd exerts protective response through the Nrf2/ARE antioxidant activation pathway in LECs as reported for other cell/tissue types. It was interesting to observe that Hyd does not induce ROS production as shown in Figure 1. Furthermore, to rule out the ROS-dependent hormetic response by Hyd, we utilized antioxidant treatment, such TAT-HA-Prdx6 protein [5,6,54] and N-acetyl cysteines (NAC), SRA-hLECs transfected with the pCAT-hPrdx6 plasmid. Then, 48 h later, the transfectants were treated with different concentrations of Hyd or cotreated with TAT-HA-Prdx6 protein (10 µg/mL) or NAC (5 mM) for 24 h. The ARE-driven CAT activity was increased in response to Hyd treatment in a dose-dependent manner (Figure 7C; gray vs. light-orange bars), while reduced activity was detected in cells transduced with TAT-HA-Prdx6 protein (Figure 7C; gray vs. green bars) or treated with NAC (Figure 7C; gray vs. blue bars). Nonetheless, SRA-hLECs were treated with TAT-HA-Prdx6 protein or NAC along with different concentrations of Hyd displayed increased *Prdx6* promoter activity. These results indicated that Hyd-mediated Nrf2 activation was ROS independent.

### 3.8. Hyd Failed to Rescue the Nrf2-Deficient SRA-hLECs against H_2_O_2_-Induced Toxicity

**Figure 8:**
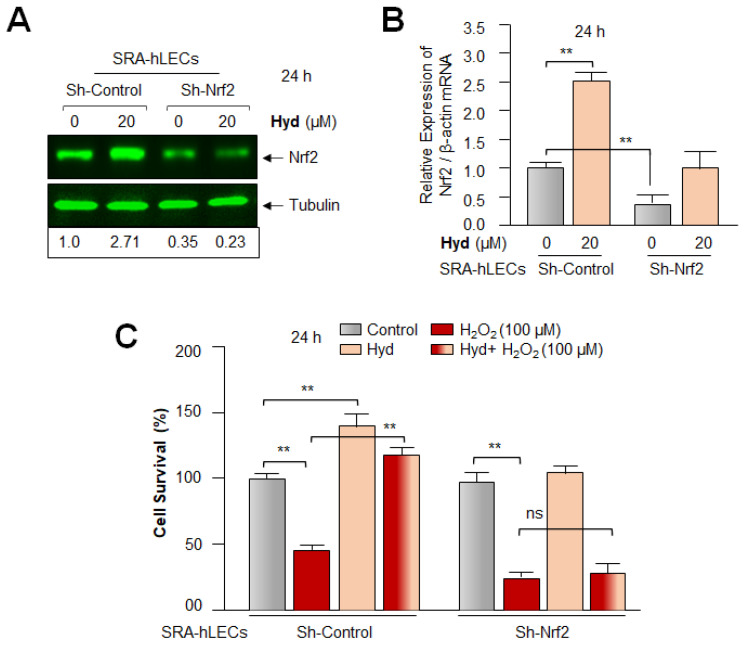
Nrf2 knockdown experiment disclosed that Hyd exerted its cytoprotective effect via Nrf2 pathway. (**A**,**B**) SRA-hLECs were transfected with either control *Sh*RNA or Nrf2 *Sh*RNA. The transfectants bearing *Sh*-Control or specific *Sh*-Nrf2 were treated with Hyd for 24 h. Total cell lysate and RNA were isolated and submitted to Western blot and RT-qPCR analyses with probes specific to Nrf2, respectively. Numbers under each protein band denote the value of Nrf2 band normalized with corresponding tubulin protein bands. The histogram data represent the mean ± S.D. values derived from three independent experiments. ** *p* < 0.001. (**C**) Nrf2 knockdown experiments demonstrated that Hyd protected LECs via Nrf2-mediated protective pathway. *Sh*-Control or *Sh*-Nrf2 SRA-hLECs as shown above in Figure 8A,B exposed to H_2_O_2_ in absence or presence of Hyd as indicated. Hyd-treated transfectants containing *sh*RNA specific to Nrf2 demonstrate a significant increase in cell death compared to *Sh*Control counterpart as evidenced by MTS assay. All histogram data represent the mean ± S.D. values obtained from three independent experiments. ** *p* < 0.001, ns; denotes nonsignificant.

To examine the impact of the Hyd-induced amplification of the Nrf2 antioxidant protective pathway, we carried out Hyd-mediated cytoprotective assay using *Nrf2*-depleted SRA-hLECs facing oxidative stress. To achieve this, we employed *Sh*Nrf2 to knock down Nrf2 and generated *Nrf2*-depleted SRA-hLECs. Expression assays analyses, Western blot (Figure 8A), and RT-qPCR (Figure 8B) revealed that Nrf2 could be successfully knocked down. Results of experimentation revealed that Hyd triggered a remarkable increase in the expression of Nrf2 protein and mRNA in *Sh*-Control SRA-hLECs, as shown in Figure 8A,B, compared to *Sh*-Nrf2 SRA-hLECs. To examine the Hyd-mediated Nrf2 antioxidant-dependent protective effects, *Sh*-Control (Nrf2 expressing, control) and *Sh*-Nrf2 SRA-hLECs (*Nrf2*-depleted SRA-hLECs) were exposed to H_2_O_2_ in the absence or presence of Hyd for 24 h. Cell viability (MTS assay) assessment disclosed that Hyd was significantly ineffective in protecting *Nrf2*-depleted SRA-hLECs (Figure 8C), while it could protect transfectants containing *Sh*-Control, (Nrf2 expressing hLECs), suggesting that Hyd exerts its protective effects specifically via the activation of the Nrf2/ARE antioxidant pathway.

### 3.9. Hyd Amplified Nrf2 and Its Target Expression and Protected the Primary mLECs against H_2_O_2_-Induced Death by Mitigating ROS Accumulation

**Figure 9:**
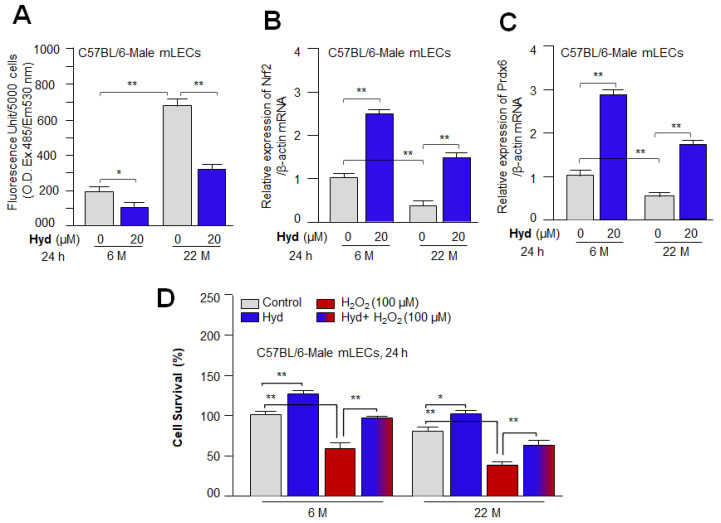
As observed in hLECs, Hyd rescued aging mLECs against oxidative stress via Nrf2-activation/reactivation antioxidant pathway. (**A**) Primary mLECs isolated from different age groups of C57BL/6 mice were cultured in 96-well plates and treated with Hyd for 24 h. Increased ROS during aging was alleviated by Hyd treatment. ROS levels were measured using H_2_-DCF-DA dye methods. The histogram data represent the mean ± S.D. value from three independent experiments. * *p* < 0.05; ** *p* < 0.001. (**B**,**C**) Primary mLECs isolated from 6 M and 22 M old C57BL/6 male mice were treated with Hyd for 24 h. Total RNA isolated from Hyd-treated primary mLECs was subjected for transcript analysis of Nrf2 and its target gene Prdx6 with specific primer sequences as shown in Table 1. All histograms represent the mean ± S.D. values from three independent experiments. ** *p* < 0.001. (**D**) Hyd was efficacious in defending both younger and aged mLECs from H_2_O_2_-induced cellular damage. Hyd treated or untreated primary mLECs were exposed to H_2_O_2_, and cell viability was assessed after 24 h. The histogram data represent the mean ± S.D. values from three independent experiments. * *p* < 0.05; ** *p* < 0.001.

Based on the above experiments, we sought to determine whether basal gene expression levels in aging primary mLECs can be reactivated by Hyd treatment and lead to cytoprotection. To achieve this, primary mLECs isolated from 6 month (M) and 22 M old C57BL/6 male mouse were treated with Hyd for 24 h. Data analysis disclosed that Hyd significantly reduced the ROS production in primary aging/aged mLECs as shown in Figure 9A. In a parallel experiment, RT-qPCR assay of the transcript levels revealed that Hyd significantly reactivated expression of Nrf2 and its target gene, Prdx6 (Figure 9B,C), demonstrating that Hyd can restore the dysregulated functions of the antioxidant pathway. However, mLECs isolated from the younger mouse were more responsive to Hyd treatment compared to the aging mouse. Next, we examined Hyd’s protective ability in defending primary LECs against H_2_O_2_-induced oxidative stress. Cell viability assessment using MTS assay (Figure 9D) showed enhanced viability of mLECs against oxidative stress in the presence of Hyd versus vehicle control (Figure 9D). Our findings underscore that FDA-approved drug Hyd should be a promising viable molecule to restore the dysregulated antioxidant cytoprotective response in aging eye lenses/LECs.

### 3.10. Hyd, When Instilled Topically in Mouse Eye, Enhanced Nrf2/ARE-Mediated Antioxidant Gene In Vivo

**Figure 10:**
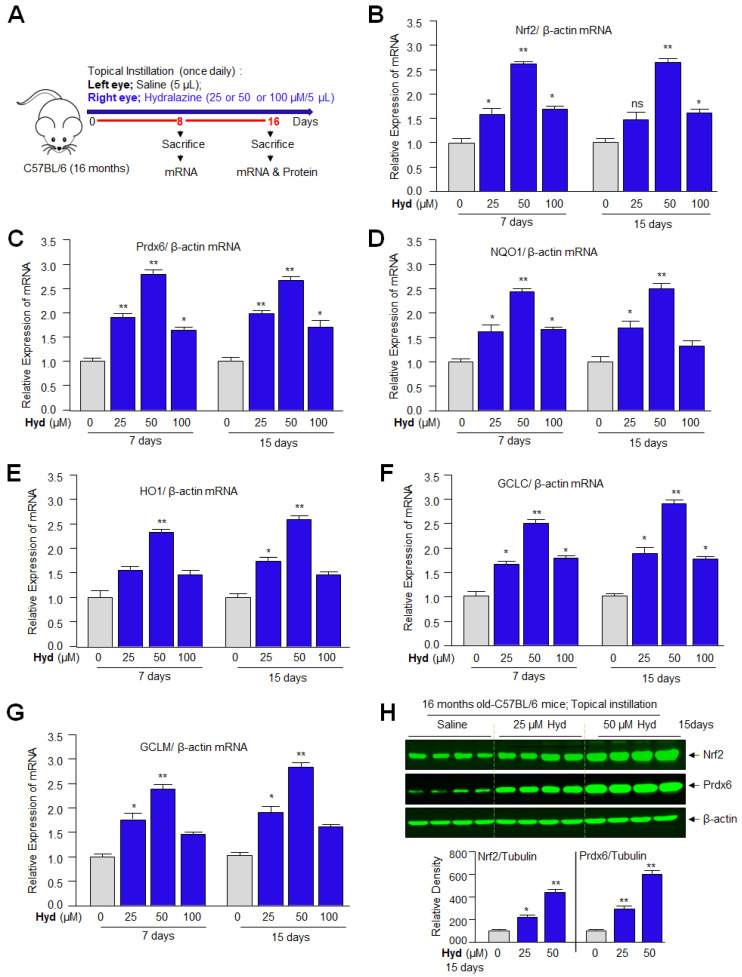
Hyd, when instilled topically in mouse eyes in vivo, enhanced Nrf2/ARE-mediated antioxidant genes. (**A**) Three groups of 16 M old C57BL/6 mice (each group; *n* = 4, 8 lenses) were used for the experiment. Hyd or buffered saline was instilled topically once daily for 7 days and 15 days as follows: Group 1, 2, and 3, vehicle control (left eye, buffered saline, pH 7.2) and Group 1, 2, and 3, Hyd treated (right eye, 25 µM/5 µL or 50 µM/5 µL or 100 µM/5 µL each eye, respectively). On day 8 or 16, mice were sacrificed, and total RNA (7 or 15-days treatment) and protein (15-days treatment) were isolated from the lenses. RT-qPCR (**B**–**G**) and immunoblotting (**H**) analyses were performed, and data were presented. (**B**–**G**) Hyd treatment significantly increased the expression of Nrf2 (**B**), and Nrf2 target antioxidant genes (**C**–**G**), as shown. The data presented as mean ± S.D. values obtained from three independent experiments. Control vs. Hyd-treated, * *p* < 0.05; ** *p* < 0.001. ns, not significant. (**H**) An equal amount of protein was loaded onto SDS-PAGE gel and immunoblotted with Nrf2, Prdx6, and β-actin antibodies, as shown. Nrf2 and Prdx6 protein bands were scanned and quantified, and the obtained values were normalized with corresponding β-actin band values. Relative densities were presented as histograms below the protein bands. * *p* < 0.05; ** *p* < 0.001.

It is evident from the above experiments that Hyd treatment activates the Nrf2/ARE-mediated protective pathway in vitro; we envisage that the results obtained from in vitro studies can be appraised if they could be reproducible in vivo. It is worth mentioning that to identify the aging-related adverse signaling linked molecular targets and to determine the protective molecular mechanism of small molecules, eye lenses/LECs have been suggested to be the best biological model system. In this study, we selected Hyd to be delivered in the form of eye drops, as this route of application will be acceptable to the subjects. In addition, we reason that this will avoid administration orally or by other routes, which may cause adverse effects in persons who do not have hypertension. Furthermore, distinct biological ages of organs have been identified, and aging of these organs have diverse genetic architectures and occurs at different rates [91,92]. In this regard, eye lenses are found to be aged faster than other organs [93], suggesting that aging is organ specific [94]. Backed by these studies, we think that the drug evaluated for its therapeutic potential to block eye lens opacity can be used for other organs’ health restoration. Thus, by using C57BL/6 mice, which have been widely used for aging research [95], herein we evaluated efficacy of Hyd’s cytoprotective effect and prevention of lens opacity in vivo/ex vivo. We have used 16 M old C57BL/6 mice (equivalent to ~52 years of human age) [96] for topical instillation of Hyd (25, 50, and 100 µM/ 5µL). The left eye of the mice was selected for topical instillation of physiological saline (pH 7.2), whereas the right eye received Hyd for 7 days or 15 days once daily as indicated in Figure 10A. On the eighth or 16th day, lenses were isolated and subjected to total RNA (Figure 10B–G) isolation to examine the Nrf2 and its major phase II antioxidant genes. RT-qPCR analysis revealed a dose-dependent increase in Nrf2’s target genes such as Prdx6, NQO1, HO1, GCLC, and GCLM at 25 and 50 µM concentrations of Hyd. However, we observed that 100 µM concentration of Hyd was not as effective as 50 µM in up-regulating the antioxidant pathway. Nrf2 mRNA expression was significantly high at 50 µM compared to 25 and 100 µM. The results revealed that Hyd could internalize in the eye lens and be biologically active as reported in other model systems. Since the RNA translation product is responsible for biological activities of genes, we next examined the cellular levels of Nrf2 and Prdx6 protein. Under the same conditions of experiments, Western blot analysis of total extract isolated from the Hyd-treated and untreated lenses of 16 M old C57BL/6 mice eyes, as shown in Figure 10A, demonstrated a relative increased abundance in Nrf2 and its target gene Prdx6, and the significant increased abundance in Nrf2/Prdx6 was dose-dependent (Figure 10H). Taken together, data revealed that 50 µM was the optimum concentration for stimulating the Nrf2/ARE signaling pathway in vivo.

### 3.11. Topical Application of Hyd Reduced the ROS Generation and Augmented Nrf2 and Its Antioxidant Genes Expression in Aged Mouse Lenses In Vivo

**Figure 11:**
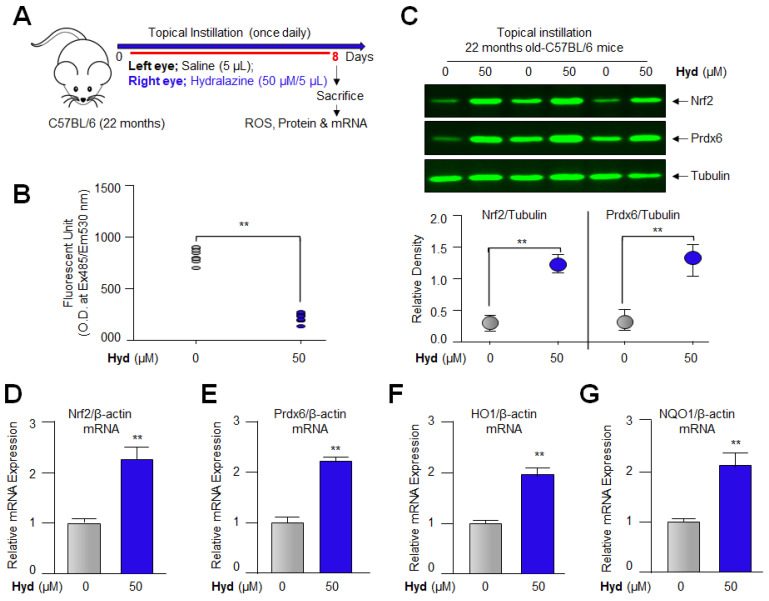

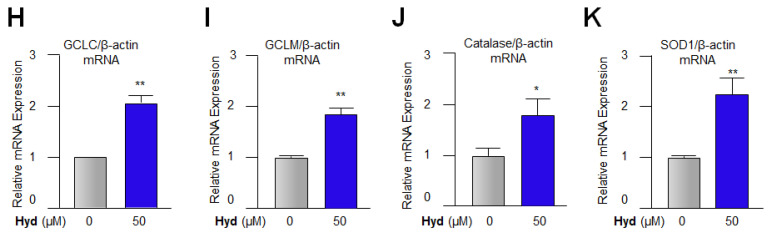
Topically instilled, Hyd enhanced expression of Nrf2 and antioxidant genes in aged mouse eye in vivo. (**A**) 22 M old C57BL/6 mice (each group; *n* = 6, 12 lenses) were used for the experiment. Hyd or buffered saline was instilled topically once daily for 7 days as follows: Vehicle control (left eye, buffered saline) and Hyd (right eye, 50 µM/5 µL in each eye). (**B**) On day 8, animals were sacrificed, and lenses were isolated and subjected to ROS quantitation using H_2_-DCF-DA dye method. The data represent as the mean ± S.D. values derived from three independent experiments. Control vs. Hyd treated, ** *p* < 0.001. (**C**) On day 8, animals were sacrificed, and total protein was isolated from lenses and subjected to Western blot analysis. Hyd enhanced the protein expression of Nrf2 and its target gene Prdx6. Tubulin was used as an internal control. Protein bands were quantified using densitometer, and levels were normalized with corresponding tubulin band intensity level. The relative density was presented in the form of histograms below the protein blots. ** *p* < 0.001. (**D**–**K**) Total RNA was isolated from the buffered saline or Hyd treated eye lenses in vivo and processed for RT-qPCR analysis. Results revealed that Hyd upregulated mRNA expression of Nrf2 (**D**) and Nrf2 target antioxidant genes (**E**–**K**). All histograms are presented as mean ± S.D. values obtained from three independent experiments. Control vs. Hyd treated, * *p* < 0.05; ** *p* < 0.001.

**Figure 12:**
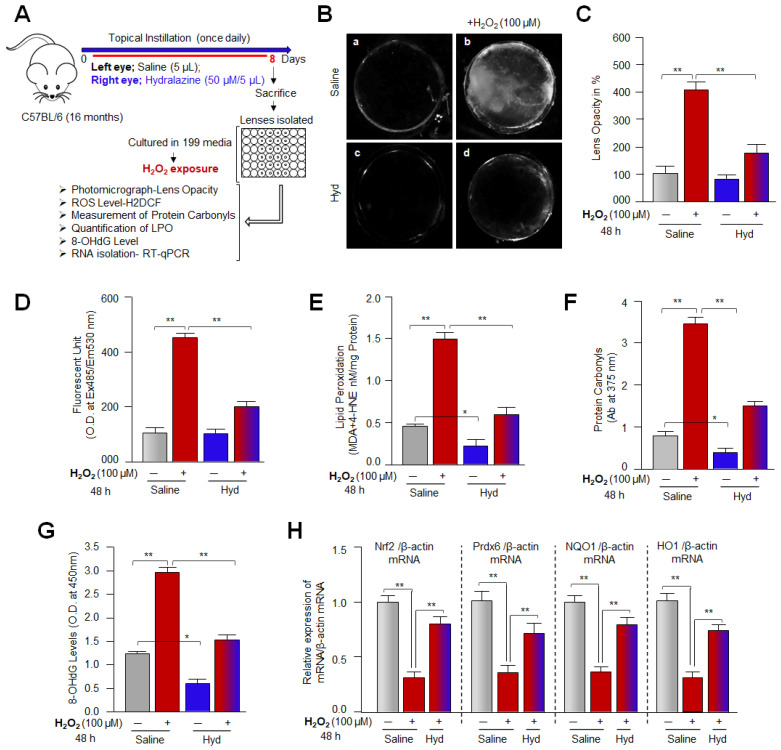
Topical delivery of Hyd in mouse eye expanded health span of lenses by delaying/preventing onset of eye lens against H_2_O_2_-induced oxidative stress. (**A**) The 16 M old C57BL/6 mice (each group; *n* = 9, 18 lenses) were used for the experiment. Hyd (50 µM/5 µL in right eye) or buffered saline (left eye) was instilled topically once daily for 7 days. On the eighth day, animals were sacrificed, isolated lenses were cultured in a 48-well plate, and exposed to H_2_O_2_ as indicated. (**B**,**C**) Eye lenses were photographed (B; a, saline treated; b, saline treated + H_2_O_2_; c, Hyd treated; d, Hyd treated + H_2_O_2_) and lens opacity was quantified with densitometric methods. Representative photographs show the effect of Hyd in delaying lens opacity facing H_2_O_2_-induced stress. The relative density of lenses was measured, and values were presented as histograms (**C**). (**D**) Under similar experimental conditions, 48 h later, lenses were homogenized, and ROS levels were quantified using H_2_-DCF-DA dye. Hyd-treated lenses showed significantly reduced ROS generation in comparison to the buffered saline-treated lenses when exposed to H_2_O_2_. (**E**) Hyd significantly alleviated lipid damage induced by H_2_O_2_. H_2_O_2_ treatment significantly increased MDA and 4-HAE levels in saline instilled lenses, which was attenuated by Hyd treatment. (**F**) Hyd prevented the H_2_O_2_-induced protein carbonylation. Protein carbonyls were measured using Protein Carbonyl Content Assay kit. Hyd application reduced the protein carbonyl levels in the lenses facing H_2_O_2_-driven stress. (**G**) Hyd effectively abated H_2_O_2_-induced oxidative DNA damage in lenses. Buffered saline or Hyd instilled lenses were cultured and treated with H_2_O_2_ for 48 h. These lenses were subjected for the quantification of 8-OHdG levels using the OxiSelect™ Oxidative DNA Damage ELISA kit. Hyd protected the lenses against H_2_O_2_-induced oxidative DNA damage. (**H**) Lenses isolated from the Hyd-instilled eye were cultured and treated with H_2_O_2_ as indicated. Then, 48 h later, total RNA was extracted and subjected to RT-qPCR for mRNA analysis of Nrf2 and its target antioxidant genes, such as Prdx6, NQO1, and HO1. (**C**–**H**) The data represent mean ± S.D. derived from three independent experiments. * *p* < 0.05; ** *p* < 0.001.

Having observed that 50 μM of Hyd was optimal for Nrf2 antioxidant pathway activation and its biological function (Figure 8 and Figure 10), we wanted to examine whether topical application of Hyd is biologically effective in aged mouse eye lenses (22 M old) (since aging-related changes or phenotypes can alter the effectivity of drug). To this end, at first, we quantified ROS levels using H_2_-DCF-DA dye method. Hyd-instilled eye lenses showed a significant reduction in ROS levels (Figure 11B) when compared to control, saline-instilled eye lenses. Next, we examined whether Hyd activated the Nrf2-ARE-mediated antioxidant pathway in 22 M old mice (equivalent to ~66 years of human age) [96]. Using the similar experimental protocol as noted in 16 M old mice (Figure 10), 22 M old mice received physiological saline (left eye) and 50 µM of Hyd (right eye) for 7 days daily. At the eighth day, total RNA and protein isolated from lenses were assessed for Nrf2 antioxidant genes expression. RT-qPCR and Western blot analyses revealed that Hyd reactivated and increased mRNA and protein expression of Nrf2 and its downstream antioxidant genes, as shown in Figure 10 and Figure 11. Nevertheless, compared to 22 M old mice, 16 M old mice were more responsive to Hyd treatment. Taken together, data revealed that Hyd has the ability to revive the Nrf2 antioxidant pathway in aging lenses, when delivered topically (as eye drop) and, thereby, amplified the antioxidant defense even in aged lenses/LECs. These findings tempted us to perform further experimentation (Figure 12) to investigate whether Hyd-treated eye lenses extend the healthspan of the lenses by preventing or delaying lens opacity against oxidative stress ex vivo.

### 3.12. Hyd’s Topical Instillation in Mouse Eye Optimized H_2_O_2_-Induced ROS Levels and Extended Lenses Health Span by Preventing/Delaying Lens Opacity against Oxidative Stress Ex-Vivo

Hyd protects LECs against H_2_O_2_-induced oxidative damage in vitro; we intended to know whether topical application of Hyd in the eye lenses in vivo could delay onset of lens opacity against oxidative stress ex vivo. To examine this, we performed in vitro lens organ culture of eye lenses topically instilled with Hyd or physiological saline and were exposed to H_2_O_2,_ as shown in Figure 12. Physiological saline (left eye) and Hyd (right eye) were topically instilled in 16 M old C57BL/6 mice eyes for 7 days. At the eighth day, isolated lenses were cultured in vitro facing H_2_O_2_ (100 µM)-induced stress. Then, 48 h later, the lenses were photographed and presented as shown in Figure 12B. In vivo Hyd-instilled lenses showed a significant reduction in lens opacity (Figure 12(Bd)) compared to the physiological saline-instilled lenses (Figure 12(Bb)). Percentage of lens opacity was presented in the form of histograms (Figure 12C). In the parallel experiments, Hyd- or physiological saline-instilled lenses in vivo were exposed to H_2_O_2_, and 48 h later the lenses were subjected to measure the levels of ROS (Figure 12D), MDA (Figure 12E), protein carbonyls (Figure 12F), 8-OHdG (Figure 12G), and mRNA (Figure 12H). Data analysis revealed a significant reduction in ROS, MDA, protein carbonyls, and 8-OHdG levels and that levels of examined molecules were directly linked to Hyd-mediated increased expression of Nrf2 and its target genes such as Prdx6, NQO1, and HO1 (Figure 12H; red bar vs. red-blue bar). Collectively, data demonstrated that Hyd could pass through the capsule of the aging lenses and internalize in LECs/lenses, as demonstrated by Hyd’s biological activity. It was intriguing to observe that Hyd extended eye lens healthspan by delaying the onset of lens opacity by enhancing Nrf2/antioxidant defense response. Results from the current study provide the proof of concept that Hyd has the ability to delay/prevent age-related cataracts.

## 4. Discussion

Increased oxidative load due to dysregulation of the Nrf2/ARE pathway is positively correlated with many pathologies and age-related degenerative diseases [97,98,99]. Recent studies reveal that activation of the Nrf2 antioxidant pathway is of great interest as a promising biological target for the pharmacological intervention to treat or prevent oxidative/age-related degenerative diseases [58,98,99,100,101]. However, clinical trial approaches on antioxidants, such as N-acetyl cysteine (NAC), Vitamin C, or E have not shown encouraging outcomes [88,102,103]. The failure of effectivity of the antioxidants can be related to their direct stoichiometric quenching of ROS. This demonstrates that cellular oxidants act through the activation of aberrant cellular signaling leading to deranged cell physiology of cells. Because Nrf2 has been found to play a pivotal role in cytoprotection against oxidative stress and aging pathobiology, it is now considered as an attractive therapeutic molecule for drug target [36,104,105,106,107]. Several compounds that target Nrf2/ARE have been tested in many oxidative-related pathobiologies; currently, at least one Nrf2 activator, dimethyl fumarate, has been approved by the US Food and Drug Administration (FDA) [108,109,110]. This suggests that cells are well equipped with a very responsive and sensitive Nrf2/ARE defense pathway [2,3,10,19]. Recent substantial studies have documented the comprehensive effect of Nrf2 activators to control or treat a variety of pathologies. In the view of the fact that the Nrf2 antioxidant pathway has been found to decline with aging, leading to increased oxidative stress-induced accumulation of protein adducts, such as carbonylation, it is a cause for onset of several disorders [22,89,111,112,113,114,115,116,117,118,119]. We, therefore, intended to rediscover the existing FDA approved drug(s) having scavenging carbonyls and Nrf2 antioxidant activation properties. In this scenario, we selected Hyd, a clinically proven “broad spectrum” scavenger of lipid-derived electrophiles (LDEs) and a potential activator of the Nrf2/ARE pathway [22,67,69,88,89].

The protective efficacy of Hyd has never been evaluated in eye lens/LECs, where dysregulation of Nrf2-ARE with oxidative stress amplification has been found to be a cause for onset of cataractogenesis [30,99,118]. In this study, we showed for the first time that Hyd is remarkably efficacious at protecting aging mouse or human LECs against oxidative stress (Figure 1 and Figure 9). We observed that application of Hyd significantly reduced the ROS levels with decreased MDA/4-HNE accumulation and DNA damage in LECs (Figure 2). These results indicated that Hyd has a great potential to rescue LECs against oxidative stress and could be as effective as reported for other cell types [21,22,88,89]. Our work also showed that Hyd treatment dramatically reduced oxidative stress-induced protein carbonyl levels (Figure 2). Protein carbonylation has been found to be a major culprit for initiation/progression of several aging pathobiologies, including cataractogenesis [113,114,115,118,120]. Furthermore, environmental and cellular stresses lead to the accumulation of ROS in a variety of organs, including the lens, and thereby increases the risk of disease onset. Oxidative stress is an initiator of lipid peroxidation (LPO) propagation, leading to aldehydes formation, such as MDA and 4-HNE, during aging. Both are electrophilic and can adduct to nucleophilic proteins, yielding to protein cross-linking, denaturation, and aggregation [112,119,121]. Degenerative diseases involving protein damage by electrophilic toxicants and oxidative stress are extensive, such as cardiovascular and neurological disorders as well as blinding diseases, including lens opacity [65,122,123]. Recent studies of Hyd’s protective effectivity in animals and invertebrate models showed that Hyd has life and healthspan extending properties [21,22,69,88,89]. As whole, studies demonstrate that in LECs, Hyd exerts its cytoprotective activity by abating ROS-induced oxidative DNA and protein damage (Figure 2), as reported earlier in other model systems [21,22,70,89,124].

Moreover, the essential question raised by our data is how Hyd delivery to cells/tissues results in ROS mitigation. To understand the molecular mechanism(s) involved in Hyd-mitigated ROS levels, our data revealed that Hyd suppresses intracellular ROS levels. This rules out the involvement of a ROS-dependent hormetic mechanism [2,3,10] (Figure 1E,F; gray vs. light orange bar) in induction of the antioxidant pathway [2]. Furthermore, Nrf2 is an authentic stress-responsive factor that transcriptionally activates antioxidant genes by binding to ARE sequences of the gene promoters [125]. Nrf2 has now been defined as a multiorgan protector, and it also blocks progression of aging/oxidative stress-associated disorders, including cataracts [36,98,99,125]. Based on these studies, including our own published reports (and as described in the Introduction Section), we surmised that protective activity exerted by Hyd could be attributed to its activation of the Nrf2/ARE antioxidant pathway. Indeed, our data derived from transactivation experimentation with engineered promoter plasmid containing ARE sites [3,82] revealed that Hyd treatment significantly increased the Nrf2/ARE-driven promoter activity [126]. Notably, the increased transcription was corroborated with increased expression of Nrf2 and major phase II antioxidant genes (such as Prdx6, NQO1, HO1, GCLC, and GCLM) (Figure 3 and Figure 4). Surprisingly, we observed that the antioxidants examined showed significantly increased enzymatic activity in Hyd-treated LECs (Figure 5). In this case, one possible mechanism for the observed increase in the antioxidant enzymes activities can be related to conformational changes of antioxidants. Nonetheless, how Hyd promoted enzymatic activity of antioxidants warrants further investigation. Taken together, our data disclose that Hyd acts via activating the Nrf2-mediated antioxidant pathway. In this support, we found that Hyd treatment increased nuclear accumulation of Nrf2 and augmentation of its target antioxidant genes expression but without affecting cellular levels of Keap1. This molecular phenomenon suggests that Hyd-mediated nuclear accumulation of Nrf2 could be independent of Keap1 activity. We observed that Nrf2 in the nuclear extract of Hyd-treated LECs was transcriptionally active (Figure 6 and Figure 7). These findings of our study suggest that Hyd may activate Nrf2 at posttranslational levels and rule out any role of ROS in regulation of Nrf2/Keap1-mediated up-regulation of antioxidant genes. However, we firmly believe that further investigation is required to unveil the molecular mechanism involved in Hyd-mediated activation of the Nrf2 pathway in lens/LECs. Nevertheless, recently, Dehghan and colleagues [21] have reported that Hyd activation of Nrf2 is a PKA-mediated mechanism. Additionally, it has been reported that CAMP/PKA and SIRT1 are upstream activators of the Nrf2/ARE antioxidant pathway in fasting mice and human hepatocytes [127]. Hyd belongs to the non-electrophilic activator of Nrf2, and it is possible that Hyd can disrupt interaction(s) between Nrf2 and its regulatory interacting protein, Keap1 [128]. Another possibility is that Hyd can target other signaling networks known to be involved in Nrf2-dependent pathways, such as the GSK-3β-TrCP, synoviolin, NF-*κ*B, Notch, or AMP kinase pathways [129]. Alternatively, it has also been suggested that it can be attributed to Hyd’s scavenging property. Hyd-trapped LDEs adducts on Keap1 may offer the protein vulnerability to Cul3-mediated degradation and thus promote Nrf2 release, thereby allowing Nrf2 to accumulate in the nucleus [130]. Although these studies support plausible mechanism(s) of Hyd-mediated regulation of the Nrf2/ARE antioxidant activation pathway, further work is warranted to explain the many Hyd-mediated beneficial effects in context to mammalian cell health.

Recently, a comprehensive body of experimental work reveals that the protective activity of Hyd is mainly attributed to its ability to amplify the Nrf2-mediated antioxidant pathway and its carbonyl scavenging property [21,67,69,70,124]. Our works also showed that Hyd protects LECs by activating the Nrf2 antioxidant pathway as well as by reducing the levels of carbonyl adducts (Figure 2, Figure 4 and Figure 5). In addition, our results demonstrated that Hyd can correct and reactivate the antioxidant pathway in aging LECs, suggesting that Hyd is a perfect viable reagent to rescue aging LECs against different kinds of oxidative stress-induced program cell death. Furthermore, emerging studies have shown that in lens/LECs, aging and oxidative stress can induce various types of program cell death, such as apoptosis, pyroptosis, and ferroptosis [6,51,131,132]. It has been established that these program cell deaths are linked to the onset of cataractogenesis [133,134,135,136]. It is worth mentioning that in vitro experiments conducted with Hyd at concentrations of 20 to 50 µM appear to be pharmacologically and physiologically relevant. Nonetheless, several in vitro studies have been conducted with higher doses of Hyd [71,124]. It has been found that at concentrations of 50 to 500 µmol/L, Hyd can exert its beneficial biological effects in endothelial and fibroblast cells [71,126]. Moreover, we observed that Hyd has some extent of similarity with metformin or resveratrol in providing a beneficial effect by activating the Nrf2 antioxidant pathway and mimicking dietary restriction [56,137]. Nonetheless, there are apparent differences between them regarding mode of action in enhancing cell/organ healthspan as well as lifespan [138,139,140]. Interestingly, compared to metformin’s doses required for activating beneficial signaling, Hyd exerts its beneficial effects at significantly lower concentrations, reducing the chances of contraindications and off-target shooting effects. This gives the advantage over other Nrf2 inductors, which are particularly critical for long-term treatment of the drug for clinical settings and application.

One of the most important findings of our present study is that topical instillation of Hyd in mouse eyes could efficiently up-regulate the Nrf2 antioxidant pathway in the lenses/LECs (Figure 10), resulting in prevention of lens opacity ex vivo. Data showed that treated lenses displayed reduced ROS levels with a significant reduction in the levels of MDA/4-HNE and protein carbonyl (Figure 12). These findings are in agreement with previously published studies, documenting that Hyd exerts its protective activity through activation of the antioxidant pathway and carbonyl adducts scavenging [21,22,69,88,124]. Furthermore, assessment of oxidative stress-induced lens opacity in relation to age-related cataracts has shown that LPO-driven cross-linking of proteins and proteins carbonylation are linked to cataract formation [118]. The aberrant levels of protein carbonyl and MDA and 4-HNE adducts have been proposed to be a marker for the protein redox status and onset of lens opacity [141,142]. Collectively, the results from our study showed that the Nrf2/ARE pathway is a safeguard of the lens, and its reactivation by means of topical application of Hyd in eyes can maintain lens clarity against oxidative stress. The results revealed that topically instilled 50 µM of Hyd in mouse eyes were significantly cytoprotective and extended lens’ healthspan. Backed with these findings, we concluded that Hyd is a potent protector of the eye lens via two mechanisms, (i) reinforcement of the Nrf2/ARE protective pathway and (ii) blunting of oxidative damage of protein and DNA. Furthermore, Hyd has strong affinity for several cells and tissues [71], and it is possible that Hyd can reach millimolar active concentrations to eye lenses via passing through the lens capsule (being a small molecule) as evidenced by its biological beneficial activity (activation of the Nrf2/ARE pathway and reduction in the protein adducts). Thus, accumulative studies derived from a variety of diverse animal and cell culture-based experimental systems, including our own results and previous reports, strongly support the potential for the repurposing of Hyd to prevent/block aging-related pathologies responsible for disease conditions. It has been shown that Hyd, whether it is delivered in vitro or in vivo/ex vivo, is highly efficacious to delay or prevent oxidative and aging-connected pathological states [21,22,67,69,70,88,89,124,143,144,145,146]. We think that due to the health promoting functions of Nrf2 [30,62,98,99,147], identification of Nrf2-activating small molecular compounds with a carbonyl scavenging property, such as Hyd, has a high potential for use as a therapeutic molecule to combat aging-associated disorders.

## 5. Conclusions

**Figure 13:**
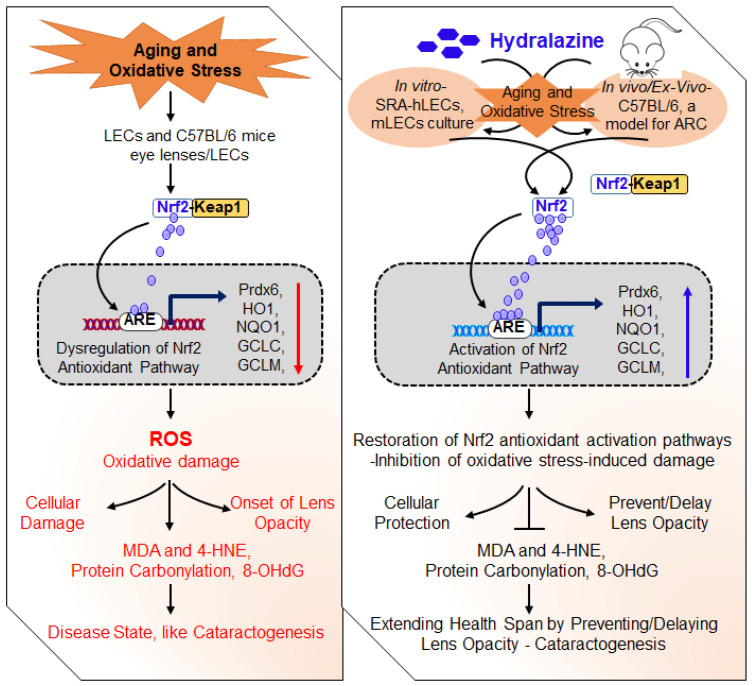
(**Left panel**) Schematic illustration displaying the effects of oxidative stress/aging on the dysregulation of Nrf2 pathways and their consequences at molecular and cellular levels. Aging lenses/LECs or lenses/LECs facing oxidative stress bear reduced levels of antioxidant expression due to dysregulated Nrf2 antioxidant pathways, which cause increased ROS and oxidative damage. Increased ROS levels result in the oxidative-driven lipid, protein, DNA, and cellular damage and in turn lead to lens opacity/cataracts (a disease state). (**Right panel**) Illustration of the molecular mechanism(s) of Hyd-mediated restoration of dysregulated the Nrf2-dependent antioxidant protective pathway, thereby leading to cellular protection and extension of lens healthspan and prevention of lens opacity in response to oxidative stress, in vitro and in vivo/ex vivo. Hereby, we propose that blinding diseases related to oxidative stress or aging can be delayed or prevented by means of Nrf2 activators, such as Hyd.

In conclusion, we reported for the first time, using in vitro and in vivo/ex vivo experiments with multiple LECs derived from mouse and human lenses along with eye lenses, that Hyd regulates redox signaling in favor of cell/organ health by activating the Nrf2 antioxidant pathway. Our data disclosed that Hyd halts ROS amplification-mediated protein or DNA damage, as well as suppresses the levels of carbonyl products, implicated for the onset of aging pathologies. This beneficial molecular event resulted in increased cellular protection and eye lens healthspan by preventing/delaying lens opacity. Furthermore, we established that the mechanism(s) driving the changes in antioxidant gene expression was related to Hyd-induced nuclear accumulation of Nrf2 and, thereby, functional enrichment of Nrf2 at ARE sequences of the antioxidant gene promoters, such as Prdx6; however, it will be necessary to unravel the exact molecular events and pathways by which Hyd promotes and regulates these changes with the involved mechanistic function of Hyd on the Nrf2 nuclear localization and antioxidant pathway activation. As Hyd is a proposed antiaging compound and effective at lower concentrations, thereby avoiding off-targets compared to other known Nrf2 activators, it is tempting to propose that Hyd deserves to be considered as a viable therapeutic molecule to ablate oxidative-/aging-associated pathogenesis and disease conditions (Figure 13).

## Figures and Tables

**Table 1:** RT-qPCR primer sequence.

Gene	Forward: 3′ to 5′	Reverse: 3′ to 5′
*hNrf2*	TGCTTTATAGCGTGCAAACCTCGC	ATCCATGTCCCTTGACAGCACAGA
*hPrdx6*	GCATCCGTTTCCACGACT	TGCACACTGGGGTAAAGTCC
*hHO1*	GGCAGAGGGTGATAGAAGAGG	AGCTCCTGCAACTCCTCAAA
*hNQO1*	ATGTATGACAAAGGACCCTTCC	TCCCTTGCAGAGAGTACATGG
*hGCLC*	ATGCCATGGGATTTGGAAT	GATCATAAAGGTATCTGGCCTCA
*hGCLM*	GACAAAACACAGTTGGAACAGC	CAGTCAAATCTGGTGGCATC
*hβ-actin*	CCAACCGCGAGAAGATGA	CCAGAGGCGTACAGGGATAG
*mNrf2*	TCTCCTCGCTGGAAAAAGAA	AATGTGCTGGCTGTGCTTTA
*mPrdx6*	TTCAATAGACAGTGTTGAGGATCA	CGTGGGTGTTTCACCATTG
*mHO1*	AGGCTAAGACCGCCTTCCT	TGTGTTCCTCTGTCAGCATCA
*mNQO1*	AGCGTTCGGTATTACGATCC	AGTACAATCAGGGCTCTTCTCG
*mGCLC*	AGATGATAGAACACGGGAGGAG	TGATCCTAAAGCGATTGTTCTTC
*mGCLM*	TGACTCACAATGACCCGAAA	TCAATGTCAGGGATGCTTTCT
*mCatalase*	CCTTCAAGTTGGTTAATGCAGA	CAAGTTTTTGATGCCCTGGT
*mSOD1*	CAGGACCTCATTTTAATCCTCAC	TGCCCAGGTCTCCAACAT
*mβ-actin*	CTAAGGCCAACCGTGAAAAG-	ACCAGAGGCATACAGGGACA

## Data Availability

Not applicable.
